# Context-Dependent Role of Vinculin in Neutrophil Adhesion, Motility and Trafficking

**DOI:** 10.1038/s41598-020-58882-y

**Published:** 2020-02-07

**Authors:** Zachary S. Wilson, Hadley Witt, Lauren Hazlett, Michael Harman, Brittany M. Neumann, Andrew Whitman, Mohak Patel, Robert S. Ross, Christian Franck, Jonathan S. Reichner, Craig T. Lefort

**Affiliations:** 1 Graduate Program in Pathobiology, Brown University, Providence, RI 02912 USA; 2 Department of Surgery, Division of Surgical Research, Rhode Island Hospital, Providence, RI 02903 USA; 3 School of Engineering, Brown University, Providence, RI 02912 USA; 4 University of California San Diego, School of Medicine, Department of Medicine/Cardiology, La Jolla, CA 92093 USA; 5 Department of Mechanical Engineering, University of Wisconsin-Madison, Madison, WI 53706 USA

**Keywords:** Inflammation, Innate immunity

## Abstract

Neutrophils are innate immune effector cells that traffic from the circulation to extravascular sites of inflammation. β2 integrins are important mediators of the processes involved in neutrophil recruitment. Although neutrophils express the cytoskeletal protein vinculin, they do not form mature focal adhesions. Here, we characterize the role of vinculin in β2 integrin-dependent neutrophil adhesion, migration, mechanosensing, and recruitment. We observe that knockout of vinculin attenuates, but does not completely abrogate, neutrophil adhesion, spreading, and crawling under static conditions. However, we also found that vinculin deficiency does not affect these behaviors in the presence of forces from fluid flow. In addition, we identify a role for vinculin in mechanosensing, as vinculin-deficient neutrophils exhibit attenuated spreading on stiff, but not soft, substrates. Consistent with these findings, we observe that *in vivo* neutrophil recruitment into the inflamed peritoneum of mice remains intact in the absence of vinculin. Together, these data suggest that while vinculin regulates some aspects of neutrophil adhesion and spreading, it may be dispensable for β2 integrin-dependent neutrophil recruitment *in vivo*.

## Introduction

Neutrophils are leukocytes of the innate immune system that are the first to respond and mobilize to sites of infection or injury. The recruitment of neutrophils from the circulation is mediated by β2 integrins that interact with endothelium adjacent to an inflamed tissue site^1,2^. Humans that lack β2 integrins or their activators suffer from leukocyte adhesion deficiency type I and III, respectively, which greatly increases host susceptibility to bacterial and fungal opportunistic pathogens^3^. However, excess recruitment and retention of neutrophils at sites of inflammation can also lead to bystander injury through the release of reactive oxygen species and proteolytic enzymes from preformed granules^4^. Many investigators have studied the mechanisms of neutrophil migration to find therapeutic targets that might enable tighter control over the inflammatory response without impairing host defense. With this long-term goal in mind, this study probes the role of vinculin in neutrophil adhesion, motility, and trafficking mediated by β2 integrins.

In the classical model of neutrophil recruitment, expression of P- and/or E-selectin on inflamed endothelium mediates the initial tethering and rolling of neutrophils. During rolling, neutrophils receive activation signals via engagement of P-selectin glycoprotein ligand-1 (PSGL-1) and G protein-coupled receptors, such as the canonical neutrophil chemokine receptors for IL-8 (human) or CXCL1 (murine). In a process called “inside-out” activation, these signals trigger structural changes in β2 integrins that increase their ligand-binding affinity by up to four orders of magnitude^5,6^. High affinity β2 integrins, primarily LFA-1 (CD11a/CD18), mediate the transition of rolling neutrophils to arrest and firm adhesion by binding to intercellular adhesion molecule-1 (ICAM-1)^7^. Finally, arrested neutrophils will then spread and crawl toward a favorable site for transmigration^8^. In this way, β2 integrin inside-out signaling mediates the steps leading to neutrophil arrest, while β2 integrin “outside-in” signaling downstream of ICAM-1 engagement is critical for stabilizing adhesion, intraluminal spreading and crawling^9^.

Vinculin is a scaffolding protein involved in the maturation of integrin-based focal adhesions that has been studied primarily in mesenchymal cells such as fibroblasts. Vinculin has multiple binding surfaces to enable the recruitment of proteins to adhesion sties^10^. Vinculin stabilizes integrin adhesions within a mature focal adhesion through the recruitment of actin-binding proteins (e.g., talin) and the direct binding of actin bundles^11^. Vinculin-dependent focal adhesion maturation has been described as being “mechanosensitive,” which refers to the recruitment of vinculin through actomyosin-mediated contractility and transmission of signals that scale with the mechanical stiffness of the substrate^12^. The current study establishes a mechanosensitive role for vinculin during neutrophil adhesion and spreading mediated by β2 integrins.

Although vinculin function has been studied in other leukocytes^13–16^, this is the first study to investigate the potential role for vinculin in leukocyte trafficking. Activated neutrophils express vinculin and form focal complexes, but they are also highly motile amoeboid-like cells that do not generate mature focal adhesions^17,18^. Adhesion stabilization enables neutrophils to crawl toward favorable sites of emigration, but whether this process is vinculin-dependent is unclear^19^. We report that while vinculin contributes to neutrophil adhesion to ICAM-1 in the absence of shear stress, it is dispensable for neutrophil adhesion and motility under shear stress, and for infiltration into the inflamed peritoneum of mice. In addition, we show that, as in other cell types, vinculin plays a role in the ability of neutrophils to sense and respond to substrate rigidity, as measured by their spread area and traction force generation. Together, these data point towards a less prominent role for vinculin in neutrophils, as compared to mesenchymal cells, that depends on the properties of the extracellular microenvironment.

## Results

### Derivation of neutrophils from conditionally-immortalized progenitors

Neutrophils are terminally differentiated leukocytes with a limited lifespan and therefore not amenable to genetic manipulation for *in vitro* studies. To circumvent this limitation, we first established the utility of HoxB8-conditional murine myeloid progenitors as an *in vitro* source of differentiated mature neutrophils. For all HoxB8-conditional progenitor cell lines used in this study, differentiation in the presence of stem cell factor (SCF) and granulocyte colony-stimulating factor (G-CSF) resulted in the complete loss in expression of CD117 (cKit) and the gain in the expression of the neutrophil-specific marker Ly6G (Supplementary Fig. S1a). LFA-1 and Mac-1, the two primary β2 integrins expressed by neutrophils, consist of the common β2 subunit CD18 and α subunits CD11a and CD11b, respectively. We observed similar expression of CD11a and CD11b by progenitor-derived wild-type (WT) and vinculin knockout (VclKO) neutrophils created using two distinct sgRNAs, and by bone marrow (BM) neutrophils isolated from mice (Supplementary Fig. S5a,b). Surface expression of CD11a and CD11b was ablated in β2 integrin knockout (Itgb2KO) neutrophils, as previously characterized^20^. All groups of progenitor-derived neutrophils expressed the canonical chemokine receptor CXCR2 (Supplementary Fig. S5c). In examining the activation of neutrophils, the upregulation of CD11b from delivery of intracellular granule stores to the cell surface was found to be similar in both WT and VclKO neutrophils, with an approximate 4-fold increase in expression in response to formylated peptide Met-Leu-Phe (fMLP) (Supplementary Fig. S5d). Altogether, these data establish the *in vitro*-derivation of genetically-modified murine neutrophils from conditionally-immortalized progenitors.

### Vinculin deficiency impairs neutrophil adhesion and spreading

**Figure 1:**
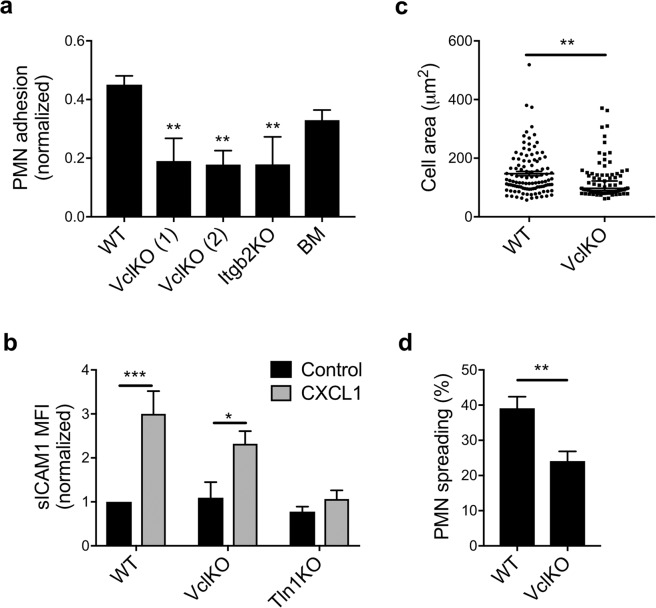
Vinculin knockout attenuates β2 integrin-dependent neutrophil adhesion. (**a**) Adhesion of neutrophils to immobilized ICAM-1 and CXCL1 assessed for progenitor-derived wild-type (WT), vinculin knockout (VclKO) created using sgRNAs (1) and (2), β2 integrin knockout (Itgb2KO), and murine bone marrow neutrophils (n = 3 independent experiments). Analyzed using one-way ANOVA with Tukey pairwise multiple comparison test. **p < 0.01. (**b**) Soluble ICAM-1 binding to neutrophils in response to CXCL1, as measured by flow cytometry (3 replicates per group, n = 3 independent experiments). Analyzed using two-way ANOVA with Tukey pairwise multiple comparison test. *p < 0.05; ***p < 0.001. (**c**) Spread area of membrane-labeled neutrophils on immobilized ICAM-1 and CXCL1 (n > 70 cells/group, n = 2 independent experiments). Analyzed using Mann-Whitney Rank Sum Test. **p < 0.01. (**d**) Percent of neutrophils spreading on immobilized ICAM-1 and CXCL1 after 30 minute incubation (n > 17 fields of view/group, n = 3 independent experiments). Analyzed using unpaired Student’s t-test. **p < 0.01.

To determine whether vinculin plays a role in β2 integrin-mediated adhesion, a static adhesion assay was used to measure neutrophil attachment to a substrate of ICAM-1 and CXCL1. We observed that vinculin-deficient neutrophils had attenuated adhesion compared to WT neutrophils, and had comparable levels of adhesion as neutrophils lacking β2 integrin expression (Fig. 1a). Adhesion levels of progenitor-derived WT neutrophils were not statistically different from that of BM neutrophils (Fig. 1a). The reduction in adhesion of vinculin-deficient neutrophils was observed over a range of substrate ligand concentrations and assay wash stringency (Supplementary Fig. S6a–c). Overall, these results suggest that vinculin plays a role in β2 integrin-mediated adhesion by neutrophils.

The activation of β2 integrins is a prerequisite to neutrophil adhesion and spreading. As an assay specific for the detection of β2 integrin affinity changes, soluble ICAM-1 binding has been previously used to demonstrate the essential role of talin-1 and Kindlin-3 in this process^6^. As VclKO neutrophils had impaired adhesion relative to WT, we sought to determine whether vinculin plays a role in the inside-out activation of β2 integrins by measuring soluble ICAM-1 binding in response to CXCL1. As expected, WT neutrophils exhibited a significant increase in soluble ICAM-1 binding in the presence of CXCL1, while talin-1-deficient neutrophils were unable to activate their β2 integrins to bind soluble ICAM-1 (Fig. 1b). Vinculin-deficient neutrophils responded to CXCL1 in a similar manner as WT neutrophils and bound significantly more ICAM-1 than unstimulated neutrophils, indicating that β2 integrin activation remains intact in the absence of vinculin (Fig. 1b). Levels of CXCL1-induced soluble ICAM-1 binding by VclKO neutrophils were not significantly different from WT neutrophils (Fig. 1b). These data indicate that while vinculin regulates neutrophil adhesion, it is not involved in the earliest steps of adhesion that rely on inside-out β2 integrin activation.

The spreading of adherent cells involves integrin clustering and actin rearrangements. To understand whether vinculin plays a role in neutrophil spreading, we quantified cell area and frequency of spreading under the same conditions as those used in evaluating neutrophil adhesion. We observed that spreading is impaired in VclKO neutrophils, in terms of both cell area and the fraction of cells that spread beyond the diameter of a round neutrophil in suspension (Fig. 1c,d). Thus, vinculin is required for efficient and complete neutrophil spreading on ICAM-1 in response to CXCL1.

### The role of vinculin in neutrophil migration

**Figure 2:**
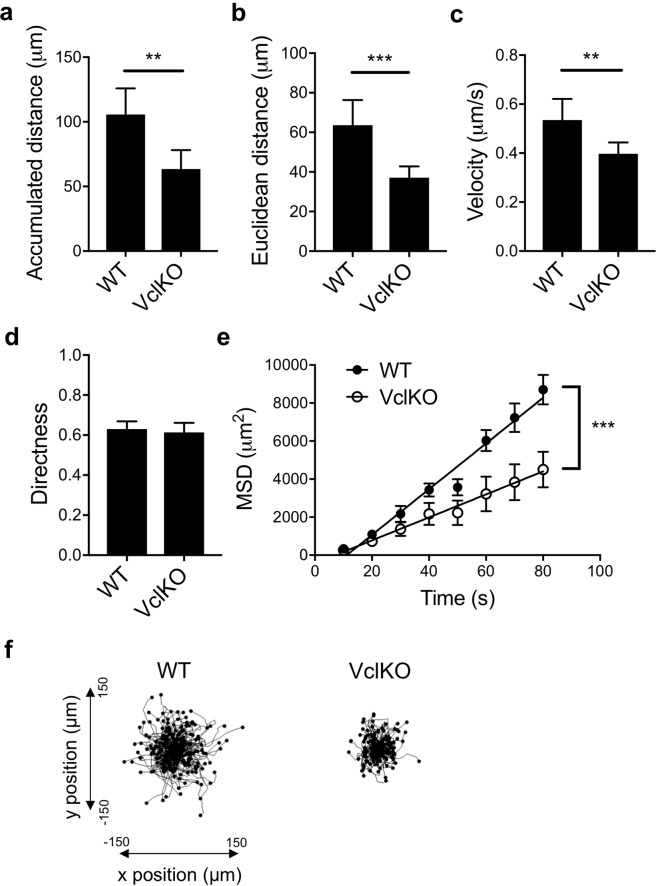
Vinculin plays a role in β2 integrin-dependent neutrophil motility. (**a**–**d**) Parameters of neutrophil motility during 30-minute chemokinesis on immobilized ICAM-1 and CXCL1 (n > 160 cells/group, 3 independent experiments). Analyzed using unpaired Student’s t-test. **p < 0.01; ***p < 0.001. (**e**) Mean-squared displacement of n**e**utrophil migration based on particle modeling. Analyzed using linear regression with comparison of slopes. WT: y = 120t − 1338; VclKO: y = 60.5t − 431.3. ***p < 0.001. (**f**) Individual neutrophil tracks during 30-minute chemokinesis on immobilized ICAM-1 and CXCL1.

Migration of neutrophils is dependent on both adhesion turnover and actin polymerization^21,22^. Here, we examined neutrophil migration to understand the potential role of vinculin in neutrophil motility during chemokinesis. As compared to WT neutrophils, VclKO neutrophils exhibited mild attenuation of migration with significantly lower accumulated distance, Euclidean distance, and instantaneous velocity (Fig. 2a–c). Directness of migration, a measure of the tendency of the neutrophil to travel in a straight line, was similar for wild-type and vinculin-deficient neutrophils (Fig. 2d). These data suggest that neutrophils generally undergo random-walk chemokinetic behavior under these conditions, as directness below 0.5 indicates less than half of migration is in the direction of its ending position. Individual neutrophil migration tracks are shown in Fig. 2f. To better analyze this behavior, a two-dimensional algorithm for measuring single particle diffusion was used to calculate mean-squared displacements for migration up to 80 seconds. Mean-squared displacement of VclKO neutrophils was significantly impaired compared to WT for migration up to 80 seconds (Fig. 2e). After 80 seconds the error for this model in both wild-type and vinculin knockout neutrophils is inflated and therefore unreliable. Altogether, these data indicate a role for vinculin in neutrophil migration under static conditions.

**Figure 3:**
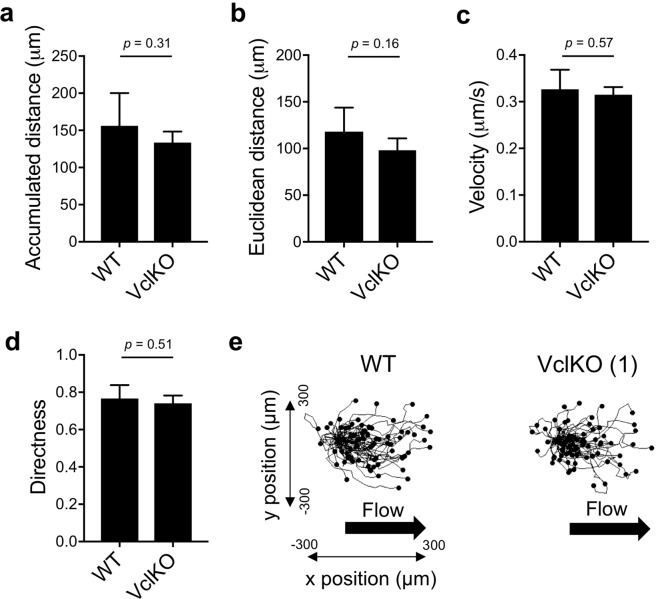
Vinculin is dispensable for neutrophil motility under shear stress. (**a**–**d**) Parameters of neutrophil motility during 60-minute chemokinesis in a flow chamber coated with E-selectin, ICAM-1 and CXCL1, and perfused at a wall shear stress of 2 dyne/cm^2^ (n > 70 cells/group, 5 replicate runs, 2 independent experiments). Analyzed using unpaired Student’s t-test. (**e**) Neutrophil tracks during 60-minute chemokinesis in a flow chamber (as in **a**–**d**).

In the context of neutrophil recruitment from the circulation, migration on ICAM-1 expressed on the endothelium will typically occur in an environment in which forces from blood flow are experienced by the attached neutrophil. Thus, to better recapitulate physiological conditions, we performed neutrophil migration assays in a flow chamber perfused at a wall shear stress within the range typical of post-capillary venules. Flow chambers were coated with E-selectin, ICAM-1, and CXCL1 to reconstitute ligands presented by inflamed endothelial cells that mediate neutrophil rolling, arrest, and intraluminal migration. In contrast to the apparent defect in VclKO neutrophil migration under static conditions, no significant difference was observed in the accumulated distance, Euclidean distance, and instantaneous velocity of WT and VclKO neutrophils in the presence of fluid shear stress (Fig. 3a–c and Supplementary Video 1). WT and VclKO neutrophil directness were similar (Fig. 3d), and the Rayleigh p-value was below 0.001 for both WT and VclKO neutrophils, suggesting that both groups of neutrophils were similarly moving in the direction of fluid flow rather than in random chemokinetic motion (Fig. 3e). Thus, in the presence of forces from fluid flow, vinculin plays no apparent role in β2 integrin-mediated neutrophil motility.

### Visualization of the actin cytoskeleton and the impact of vinculin deficiency

**Figure 4:**
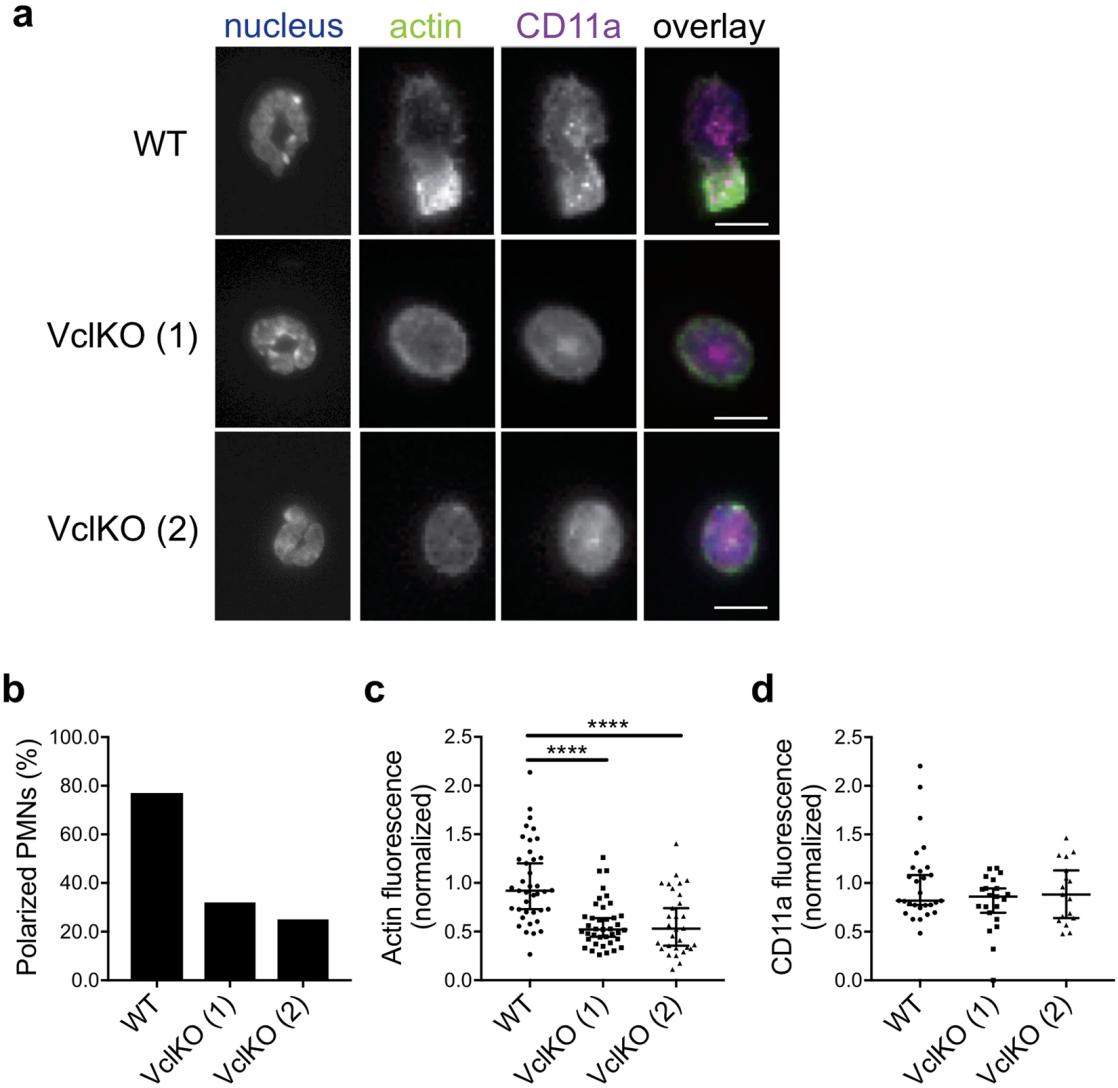
Vinculin is required for stable F-actin localization within uropods and neutrophil polarization. (**a**) Representative immunoflluorescence images of neutrophils after 30-minute incubation on immobilized ICAM-1 and CXCL1. Cells were stained with Hoescht, phalloidin, and anti-CD11a (n = 3 independent experiments). Scale bar = 10 µm. (**b**) Polarization of neutrophils based on asymmetric F-actin distribution (n = 3 independent experiments). (**c**,**d**) Background-subtracted and normalized TIRFM fluorescent intensities of F-actin and CD11a (n > 30 cells/group, 3 independent experiments). Analyzed using Kruskal-Wallis one-way ANOVA with Dunn’s multiple comparison test. ****p < 0.0001.

To understand whether the impairment in spreading and migration are related to the actin cytoskeleton, WT and VclKO neutrophils adherent to immobilized ICAM-1/CXCL1 were examined for the localization of actin by fluorescence microscopy. CD11a, the α subunit of LFA-1, was used to examine integrin localization^19^. Phalloidin was used to stain F-actin, which is expected to localize to uropods as stable F-actin fibers and as actively polymerizing in the lamellipodia during migration^22,23^. Total internal reflection fluorescence microscopy (TIRFM) was used to selectively image fluorescent signals at the cell surface interacting with the substrate. WT neutrophils had a strong localization of F-actin within uropods, where force is expected to be generated for ameboid migration^24^, while there was no discernable organization pattern of CD11a (Fig. 4a). To evaluate colocalization of F-actin and CD11a, the Pearson’s coefficient was calculated to be 0.876 for WT neutrophils, 0.859 for VclKO (1), and 0.713 for VclKO (2). The similar degree of colocalization of F-actin and CD11a between groups suggests that β2 integrins may remain able to interact with the actin cytoskeleton in the absence of vinculin. In observing actin distribution, 77% of WT neutrophils could be considered polarized compared to 32% and 25% in VclKO (1) and (2), respectively (Fig. 4b and Supplementary Fig. S7a). The fluorescent skewness and kurtosis of F-actin distribution was measured to determine whether the distribution of fluorescence was asymmetric around the mean or peaked, respectively. Skewness of greater than 1 can be considered asymmetrical while positive kurtosis indicates peaked intensity away from a Gaussian distribution. F-actin median skewness in WT neutrophils was observed to be 1.23 compared to VclKO (1) and (2), which have a median skewness of 0.89 and 1.02, respectively (Supplementary Fig. S7b). All neutrophils displayed similar median kurtosis values (Supplementary Fig. S7b). After normalizing to WT, VclKO (1) and (2) neutrophils had a median F-actin intensity in TIRFM images that was significantly less than that of WT neutrophils (Fig. 4c), whereas CD11a intensity was not different across groups (Fig. 4d). Neutrophil morphology was measured based on aspect ratio, roundness and circularity; higher values of aspect ratio and lower values of roundness and circularity imply more elongated neutrophil morphology. The median circularity, median roundness and median aspect ratio of WT neutrophils were significantly different than that of VclKO neutrophils (Supplementary Fig. S7b). Altogether these data indicate that vinculin-deficient neutrophils are less polarized compared to WT neutrophils, which may be related to impairment in actin cytoskeletal organization. When vinculin was expressed endogenously with a fluorescent tag or visualized using antibody labeling, it was found to localize to the perimeter of the neutrophil (Supplementary Fig. S8a,b), as has been previously described^18,25^. Using neutrophils expressing Clover-vinculin and Lifeact-mRuby2, to visualize actively polymerizing actin, surface-proximal vinculin was tracked in a live neutrophil migrating on ICAM-1/CXCL1 (Supplementary Fig. S8a). We observed that Clover-vinculin increased in intensity as neutrophils contracted inward during migration, suggesting a potential role for vinculin during the contraction stage of migration.

### Vinculin is dispensable for neutrophil recruitment *in vivo*

**Figure 5:**
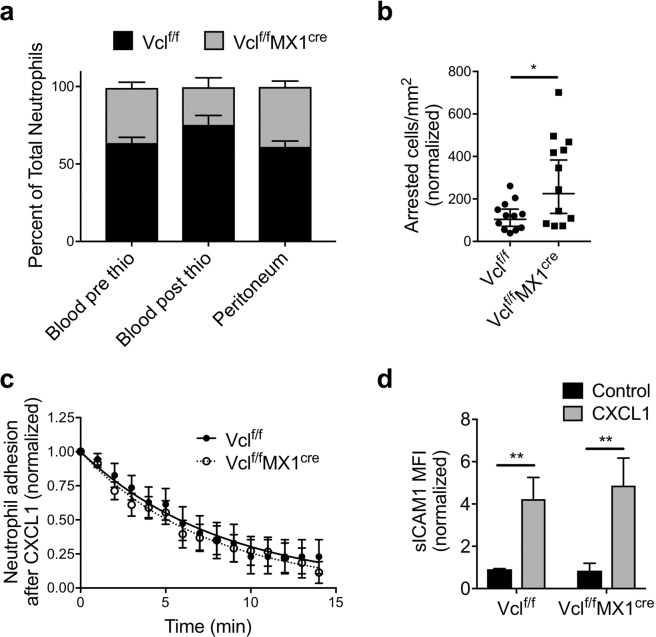
Vinculin is dispensable for neutrophil recruitment *in vivo*. (**a**) Percentage composition of control (Vcl^f/f^) and vinculin knockout (Vcl^f/f^MX1^cre^) neutrophils in the peripheral blood and peritoneal lavage, 4 hours after induction of peritonitis in mixed chimeric mice (n = 5 mice, 2 independent experiments). Analyzed using two-way ANOVA with Tukey multiple comparison test. (**b**,**c**) The arrest and time course of sustained adhesion of neutrophils in response to intravenous injection of CXCL1, and over the 15-minute period immediately following (n = 12 fields of view, across 7 chimeric mice). Data were analyzed using non-linear regression: (WT) Y = e^−0.119t^, (VclKO) Y = e^−0.136t^. (**d**) Soluble ICAM-1 binding to bone marrow neutrophils in response to CXCL1, as measured by flow cytometry (3 replicates per group, n = 3 independent experiments).

To better understand the apparent contradictory results on the role of vinculin in neutrophil migration, a murine mixed bone marrow chimeric model was used to examine whether vinculin regulates *in vivo* neutrophil recruitment. Mixed chimeric mice allow for analysis of competitive *in vivo* recruitment of wild-type (Vcl^f/f^) and vinculin knockout (Vcl^f/f^MX1^cre^) neutrophils in an internally controlled inflammatory environment (Supplementary Fig. S9a,b). For mice challenged with an intraperitoneal injection of thioglycollate broth that induces sterile inflammation, neutrophil recruitment peaks during the first 4–6 hours and occurs through mechanisms involving β2 integrins^1^. Comparing the baseline (pre-stimulus) peripheral blood chimerism to that observed in the peritoneal lavage at 4 hours after inducing peritonitis, we found no significant difference in the recruitment of wild-type and vinculin-deficient neutrophils (Fig. 5a). Additionally, *in vitro* progenitor-derived WT and VclKO neutrophils were adoptively transferred into C57BL/6 mice to observe their competitive recruitment during thioglycollate-induced peritonitis. Again, there was no difference in the recruitment of *in vitro*-derived WT and VclKO neutrophils, whereas Itgb2KO neutrophils exhibited impaired recruitment into the inflamed peritoneum as expected (Supplementary Fig. S9c,d).

The murine cremaster muscle microvasculature was observed in mixed chimeric mice by intravital microscopy during soluble chemokine (CXCL1) stimulation, which has previously been shown to induce rapid β2 integrin-mediated neutrophil arrest that is dependent on integrin activation by talin-1 and Kindlin-3^6^. In addition, the time that elapses prior to detachment of neutrophils after CXCL1-indued arrest was used to measure adhesion strengthening^26^. We observed no impairment in the arrest or adhesion strengthening of vinculin-deficient neutrophils relative to wild-type (Fig. 5b,c). Further, Vcl^f/f^ and Vcl^f/f^MX1^cre^ bone marrow neutrophils assayed *ex vivo* exhibited a similar increase in soluble ICAM-1 binding (Fig. 5d), supporting previous results using progenitor-derived neutrophils and indicating that vinculin is not required for β2 integrin activation. It is therefore unclear how vinculin deficiency results in enhanced numbers of neutrophils arresting on post-capillary venules after CXCL1 stimulation (Fig. 5b). Altogether, these *in vivo* data are consistent with our *in vitro* findings analyzing neutrophil migration under fluid flow that suggest vinculin is dispensable for intraluminal neutrophil motility. Further, these *in vivo* data also suggest that the process of neutrophil diapedesis and entry into extravascular tissue sites does not require vinculin.

### Vinculin mediates mechanotransduction during neutrophil adhesion

**Figure 6:**
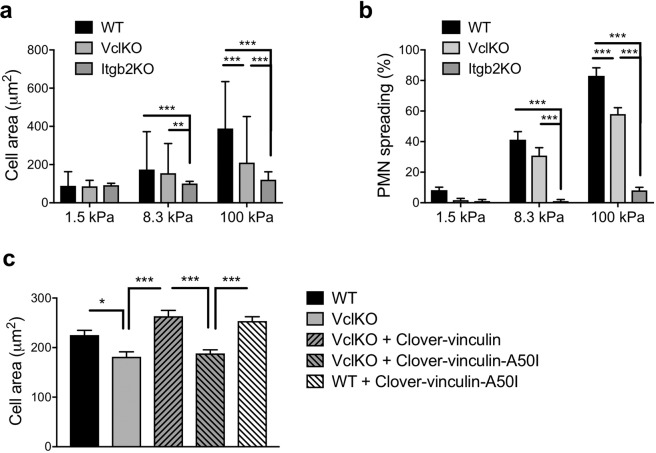
Vinculin plays a role in neutrophil mechanosensing. (**a**,**b**) Neutrophil cell area and spreading frequency on polyacrylamide gels of varying stiffness (soft: 1.5 kPa, intermediate: 8.3 kPa, stiff: 100 kPa) conjugated with ICAM-1 and CXCL1 (n > 100 cells/group, 15 fields of view/group, 3 independent experiments). Analyzed using two-way ANOVA with Tukey multiple comparison test. **p < 0.01, ***p < 0.001, ****p < 0.0001. (**c**) Area of neutrophils from the indicated groups of WT and VclKO (2) cells with exogenous expression of Clover-vinculin in wild-type or A50I mutant forms. Neutrophils were allowed to adhere and spread on 100kPa polyacrylamide gels conjugated with ICAM-1 and CXCL1 (n > 250 cells/group, 12 fields of view/group, 3 independent experiments) *p < 0.05, ***p < 0.001.

Vinculin is well characterized for its mechanosensitive function in other cell types^27,28^, and so we reasoned that vinculin may play an analogous role in neutrophils. The spreading of human neutrophils has been shown to depend on substrate stiffness^29^, but the molecules involved in the neutrophil mechanosensing response have yet to be identified. To probe the function of vinculin in neutrophil mechanosensing, we analyzed neutrophil spreading on polyacrylamide gels of varying stiffness that were functionalized with ICAM-1 and CXCL1. With increasing substrate stiffness, WT neutrophils exhibited an increase in cell area and the fraction of neutrophils that spread, whereas VclKO neutrophils exhibited an attenuated response that differed significantly from WT neutrophils only at the highest (100 kPa) substrate stiffness (Fig. 6a,b and Supplementary Fig. S10a). While the mechanosensitive spreading of VclKO neutrophils was significantly attenuated relative to WT, vinculin deficiency did not completely ablate β2 integrin-dependent spreading, as demonstrated by comparing VclKO neutrophils to Itgb2KO neutrophils lacking β2 integrin expression (Fig. 6a,b). We further probed neutrophil adhesion and motility at intermediate substrate compliance (5–20 kPa), observing that the mechanosensitive phenotype of vinculin-deficient neutrophils became measurable in our experimental system within this intermediate range of substrate stiffness (Supplementary Fig. S10b). In addition, we observed that neutrophils lacking vinculin had impaired motility on 5 kPa and 10 kPa gels, but not on 20 kPa gels (Supplementary Fig. S10c).

To gain further insight into the mechanisms of vinculin-mediated neutrophil mechanosensing and spreading, we attempted rescue of VclKO neutrophil spreading on 100 kPa substrates by exogenous expression of wild-type or mutant forms of vinculin. Expression of vinculin tagged with a variant of GFP, Clover-vinculin, was able to enhance the spread area of VclKO neutrophils to levels observed in WT neutrophils (Fig. 6c). However, expression of Clover-vinculin-A50I, with a single amino acid mutation that disrupts interaction with talin^11^, was not able to rescue the spreading deficiency of VclKO neutrophils (Fig. 6c). Altogether, these data indicate that vinculin regulates β2 integrin-dependent neutrophil spreading through a mechanosensing mechanism that involves vinculin interaction with the integrin tail-binding protein talin.

**Figure 7:**
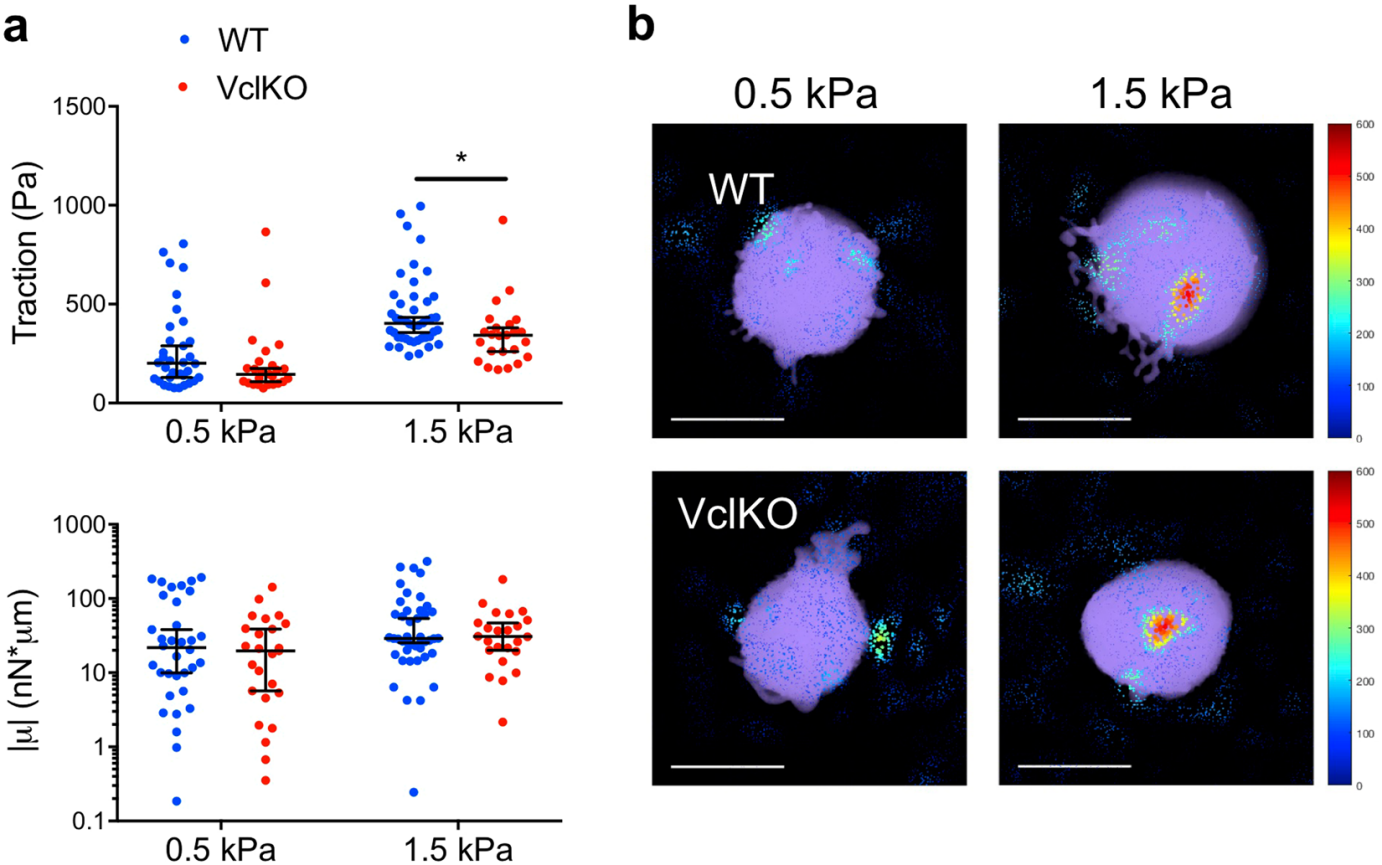
Neutrophil traction stress generation is attenuated by vinculin deficiency. (**a**) 3D tractions and the trace of the dipole moments, µ, of WT and VclKO neutrophils on polyacrylamide gels of either 0.5 kPa or 1.5 kPa stiffness (n > 25 cells/group, 3 independent experiments). Data analyzed using two-way ANOVA with Tukey multiple comparison test. *p < 0.05. (**b**) Representative 3D traction cone plots for WT and VclKO neutrophils (shown in purple) on polyacrylamide gels of indicated stiffness. Cones indicate traction direction while color and size represent traction magnitude, in Pascals. Scale bar = 10 µm.

Contractile force generation is essential for neutrophil adhesion, spreading, and migration^21^. To quantify contractility, we performed traction force microscopy using bead-embedded polyacrylamide gels. There was a technical limitation for these studies, in that only on gel substrates of relative low stiffness (less than 1.5 kPa) do neutrophils produce measurable gel/bead displacements. Nevertheless, traction force microscopy is a sensitive technique capable of resolving small differences in traction stress that do not necessarily manifest in a population-level phenotype. Polyacrylamide gels were functionalized as above with ICAM-1 and CXCL1, but with 40-fold more ICAM-1 and 2-fold more CXCL1 to maximize contractility in each individual cell. Possibly due to this increased ligand density, neutrophils underwent adhesion and spreading, but there was no observable long-range migration under any of the measured conditions. *In vitro*-derived WT and VclKO neutrophils generated increased traction stresses from very soft (0.5 kPa) to soft (1.5 kPa) gels, but had similar overall contractility under the tested conditions (Fig. 7a,b). VclKO neutrophils had reduced traction stresses on soft gels compared to WT neutrophils, but similar overall contractility for both very soft and soft gels. These data are consistent with VclKO neutrophil spreading being unimpaired on gels of lower matrix relative to WT, suggesting again that the magnitude of the role for vinculin in neutrophil adhesive function depends on the extracellular mechanical microenvironment.

## Discussion

The goal of this study was to assess the role of vinculin in neutrophil adhesion and motility. In the classic example mesenchymal cell adhesion, vinculin is involved in the maturation of integrin-based focal adhesions and contributes to cell spreading and mechanotransduction^30,31^. In leukocytes, vinculin has been shown to have roles in unique processes like T and B cell immune synapse formation, marking apoptosis for T cells, and osteoclast actin-ring formation during bone resorption^14,16,32^. In platelets, vinculin is dispensable for most physiological functions^33^. Whether vinculin is a mediator or regulator of neutrophil adhesion was uncertain prior to the current study. Although neutrophils do not form mature focal adhesions, we hypothesized that vinculin may play a role in neutrophil behavior during processes such as adhesion strengthening to the endothelium.

By live cell imaging, we observed that vinculin accumulates in punctate structures in the neutrophil as it contracts inward on ICAM-1, similar to what has been previously described for vinculin localization in neutrophils^34^. Our study finds that vinculin knockout attenuates neutrophil adhesion, spreading and migration on ICAM-1 *in vitro* under static conditions. The attenuated response in adhesion mimics the attenuation in spreading, which may imply that the neutrophils remaining adhered in the static adhesion assay are those that are spread. Despite the *in vitro* defect observed in neutrophils lacking vinculin, there was no *in vivo* phenotype as assessed in a classical recruitment model of acute peritonitis. Furthermore, neutrophil adhesion strengthening in inflamed post-capillary venules of the cremaster muscle remained intact in vinculin-deficient neutrophils. In fact, there were significantly more vinculin knockout neutrophils that underwent rapid arrest in post-capillary venules in response to CXCL1, an *in vivo* assay of β2 integrin activation. It is possible that in mixed chimeric mice the supply of vinculin knockout neutrophils to the cremaster muscle vascular bed was transiently enhanced relative to wild-type, as neutrophils can become sequestered elsewhere in the circulation in response to systemic stimuli.

During sterile inflammation, chemokines form a gradient outward from the offended site that guides migrating neutrophils^35^. Neutrophils first encounter these chemokines intravascularly as they are immobilized by heparan sulfate on the apical surface of the inflamed endothelium^35^. Neutrophil crawling on inflamed endothelium is necessary to find a favorable site to transmigrate, and adhesion strengthening aids this process as the neutrophil must resist detachment under the shear stress of blood flow^8,26,36,37^. Interestingly, we found that the involvement of vinculin in regulating neutrophil migration is dependent on the hemodynamic context in which it is assayed. While the migration of vinculin-deficient neutrophils was reduced compared to wild-type neutrophils in a commonly used static motility assay, no impairment in migration under flow was observed in vinculin knockout neutrophils. In the flow chamber assay, there was a preference for neutrophils to crawl in the direction of shear stress rather than opposing it, and both wild-type and vinculin-deficient neutrophils exhibited this directional behavior. There is much evidence for *in vivo* chemokine gradients superseding *in vivo* physical cues such as shear stress to direct neutrophils toward sites of injury^35,38^. However, shear stress is still a quantifiable factor *in vivo* with neutrophils often favoring travel with flow rather than against^36^. Addressing the chemotactic persistence of neutrophils under flow after vinculin ablation would be critical as this has been ascribed as a critical role for vinculin, which was not resolved with our experiments that employed immobilized CXCL1 as a chemokinetic stimulus^39^. In the absence of forces due to shear stress, random migration was observed, with no significant role for vinculin in the persistence of neutrophil migration based on directness and the linearity of the mean-squared displacement graph^40^. However, the model in which mean-squared displacement was calculated has a limited fit for calculations at greater time intervals, and the curvature of mean-squared displacements for wild-type and vinculin knockout neutrophils might not be completely solved.

Outside-in signaling by integrins plays a prominent role in actin cytoskeletal reorganization for physiological functions such as migration. Here we show a marked ablation of stable F-actin and neutrophil polarization on ICAM-1 in vinculin knockout neutrophils that may indicate an impaired outside-in integrin signaling response. Stable F-actin is important for the contractility of neutrophils and force generation is attenuated in vinculin knockout neutrophils as quantified by traction force microscopy. Outside-in signaling by ligand-bound integrins is thought to occur through immunoreceptor tyrosine-based activation motif (ITAM)-containing receptors, such as the low affinity Fc receptor Fcγ and DAP12 expressed in leukocytes^41^. These receptors are phosphorylated by Src family kinases (SFKs) to recruit and activate Syk, leading to downstream activity of PLCγ2, Vav exchange factors, PI3K, and SLP-76 adaptor. In this way SFKs are responsible for adhesion strengthening, morphological change, and migration through outside-in signaling. However, while Syk has been found to localize with proteins such as the β2 integrin Mac-1 (CD11b/CD18) and mAbp1, which are important for intraluminal crawling, the proteins necessary for integrin outside-in signaling (Syk, SFKs and ITAM-containing receptors) have been found to have disparate roles in intraluminal crawling and only minimally influence extravasation in fMLP- and chemokine-mediated models of neutrophil recruitment^42^. For example, a recent study found the Tec family kinase Btk and the SFK Hck, while indispensable for fMLP-mediated intraluminal crawling and recruitment, were dispensable for chemokine-mediated neutrophil recruitment^43^. Our study is limited to chemokine-mediated neutrophil recruitment, so we cannot rule out other pathways of activation that might involve vinculin-dependent intraluminal crawling or outside-in signaling in general. Interestingly, while PKCθ and mAbp1 are implicated in adhesion strengthening and intraluminal crawling under flow conditions, respectively, they have more dispensable roles under static conditions^26,36^. Here we describe the reverse, in which vinculin has a dispensable role for neutrophil crawling under shear forces but is important for crawling in the absence of flow *in vitro*. Together with these previous findings, our data suggests that neutrophils employ distinct mechanisms for migration that depend upon extracellular microenvironmental conditions and cues. One might speculate that vinculin is more important to interstitial crawling compared to vascular recruitment, but additional studies are necessary to directly address this possibility. The *in vitro* system used in our study to probe neutrophil migration is two-dimensional and used ligands to mimic endothelium, while interstitial crawling in tissues is most often three-dimensional and can have a dispensable role for integrins^44^.

Outside-in and inside-out signaling have been defined for integrins to describe their nature as both adhesion molecules and signaling receptors^2^. We find that vinculin was not required to regulate neutrophil β2 integrin activation through inside-out signaling when neutrophils were activated by soluble CXCL1. We cannot rule out that vinculin may participate in integrin regulation through an outside-in pathway once bound to ICAM-1, in which talin-1 is bound to the β2 integrin tail^6^. The outside-in component of integrin signaling was of interest to this study because of vinculin’s known role in mechanotransduction in other cell types^30,45^. As neutrophils can travel from the blood to virtually all parts of the body, sensing the mechanical environment is of immense importance to fine tune neutrophil function in different tissues^29^. In mesenchymal cells, rigidity sensing is a well-characterized adaptive response that influences focal adhesion formation, cell spreading, and traction force generation^46^. Indeed, our data indicate that vinculin can also mediate mechanosensing by neutrophils. However, the stiff gels of 100 kPa used in our study are not of a physiologically relevant tissue compliance. Vinculin-dependent spreading was only found to be impaired on 100 kPa substrates, while traction stress generation and spreading was not impaired on physiologically relevant substrate stiffnesses between 1–10 kPa. Rescue of vinculin-deficient of neutrophil spreading was achieved through exogenous expression of full-length vinculin, but not by the A50I mutant of vinculin that disrupts its interaction with talin-1. These data point towards a mechanism that involves the function of vinculin at integrin-based adhesion structures. A mechanosensing phenotype was also resolved using traction force microscopy, with a small, but significant, attenuation of neutrophil contraction observed after vinculin depletion. This defect would be unlikely to affect motility, which agrees with the *in vivo* model and suggests that physiological neutrophil motility is unimpaired in the absence of vinculin. Considering that vinculin expression is unnecessary for neutrophil recruitment using a murine model of acute peritonitis, it remains unclear whether this might be because rigidity sensing by neutrophils does not play a role in their recruitment to this specific tissue.

In conclusion, we report that the role of vinculin in neutrophil adhesive function mediated by β2 integrins is highly dependent on the mechanical context of the extracellular microenvironment. Analysis of neutrophil spreading and adhesion under static conditions, using assays commonly used in the field, first suggested a prominent role for vinculin. By performing assays of mechanosensitivity and of adhesion and migration under flow, we observed that vinculin is dispensable when experimental conditions more closely mimic physiological conditions. Finally, we found that vinculin is also not required for neutrophil recruitment in an animal model of sterile inflammation. Further studies are necessary to probe other inflammatory contexts and tissue sites to determine whether a vinculin-dependent mode of neutrophil adhesion and motility is employed *in vivo* under different microenvironmental conditions.

## Materials and Methods

### Antibodies and reagents

All antibodies used are against murine antigens. Antibodies: anti-CD18 (clone GAME-46; BD Biosciences), anti-CD11a (clone M17/4; BioLegend), anti-ICAM-1 (clone YN1; BioLegend), APC-anti-CD11b (clone M1/70; BioLegend), anti-Ly6G (clone 1A8; BioLegend), APC-anti-CD117 (clone 2B8; BioLegend), anti-CXCR2 (clone SA045E1; BioLegend), anti-α-actinin (Cell Signaling Technologies), anti-vinculin (Cell Signaling Technologies), anti-GFP (Cell Signaling Technologies), HRP-conjugated-anti-Rabbit IgG (Cell Signaling Technologies), anti-CD11a (clone IBL-6/2; Cell Signaling Techologies), Alexa Fluor 647-anti-Rat IgG (ThermoFisher Scientific). Reagents: recombinant murine CXCL1 (BioLegend), recombinant murine SCF (BioLegend), recombinant murine G-CSF (BioLegend), recombinant murine ICAM-1 (R&D Systems, BioLegend), 4-Hydroxytamoxifen (Tocris), carboxyfluorescein succinimidyl ester (CFSE, BioLegend), TagIt-Violet (BioLegend), Thioglycollate broth (Sigma Aldrich), PKH26/PKH67/Claret Far Red Membrane Dye (Sigma Aldrich).

### Neutrophil progenitors

Neutrophils were obtained by differentiating murine myeloid progenitors that were conditionally-immortalized using tamoxifen-inducible HoxB8^47^. Briefly, murine hematopoietic stem/progenitor cells were isolated from bone marrow (StemCell Technologies), transduced with a tamoxifen-inducible expression vector for the murine *Hoxb8* gene^48^, and then cultured in the presence of 100 nM 4-Hydroxytamoxifen (4-OHT), 50 ng/mL recombinant murine stem cell factor, and 1 μg/mL puromycin. Progenitors were differentiated into neutrophils by removing 4-OHT and culturing in the presence of 20 ng/mL recombinant murine stem cell factor and 20 ng/mL recombinant murine granulocyte colony-stimulating factor for 4 days (Supplementary Fig. S1a)^49^. Neutrophils differentiated from progenitors exhibit multi-lobed nuclei, expression of Ly6G, and a loss in the expression of CD117 (cKit) (Supplementary Fig. S1b,c). To create vinculin knockout progenitor cell lines for this study, HoxB8-conditional progenitors were transduced with a lentiviral vector that expresses Cas9 and single-guide RNA (sgRNA) targeting the *Vcl* gene that encodes vinculin. To do so, we used the pLentiCRISPR v2 vector, a gift from Feng Zhang (Addgene plasmid #52961) that was modified to confer blasticidin resistance, and the following sgRNA target sequences: TTCCCCTAGAGCCGTCAATG (Vcl (1)) and CCGGCGCGCTCACCCGGACG (Vcl (2)) (Supplementary Fig. S2a). The *Tln1* and *Itgb2* genes were knocked out in HoxB8-conditional progenitors as previously described^20^. Empty vector expression of Cas9 without a targeting sgRNA was used as a wild-type control for all experiments. Vinculin was successfully disrupted in progenitors after a single lentiviral transduction that was followed by blasticidin selection (Supplementary Fig. S2b). When using a fluorescent reporter of HoxB8 expression, no difference was observed between wild-type (WT) and vinculin knockout (VclKO) EGFP-HoxB8 expression before differentiation and both exhibited a similar loss of EGFP-HoxB8 expression at the end of four days of differentiation (Supplementary Fig. S2c).

For rescue studies, Clover (a GFP variant) conjugated to vinculin or the vinculin A50I mutant were cloned into the doxycycline-inducible Tet-On 3 G plasmid system (Takara Bio). HoxB8-conditional progenitors (expressing Cas9 and control sgRNA or Vcl (2) sgRNA) were transduced by lentivirus with the inducible Clover-vinculin constructs and then treated with doxycycline (Sigma Aldrich) at a concentration of 1 μg/mL to induce expression of Clover-vinculin. After transduction, progenitors were sorted for high expression of Clover-vinculin using fluorescence activated cell sorting (FACS). Clover-vinculin was successfully expressed in both control and VclKO progenitors derived from Vcl (2) sgRNA that targets the intron-exon junction, and therefore does not target exogenous Clover-vinculin (Supplementary Fig. S3).

### Neutrophil static adhesion assay

Neutrophils obtained after 4-day differentiation (“*in vitro*-derived”) were washed and labeled using CFSE (BioLegend). Murine bone marrow neutrophils were isolated by negative selection (StemCell Technologies) and immediately labeled alongside *in vitro*-derived neutrophils using CFSE. 96-well plates were coated for 1 hour at room temperature or overnight at 4 °C with 2.5, 5, or 7.5 µg/mL ICAM-1 and/or 2.5 µg/mL CXCL1 in phosphate buffered saline (PBS) and then blocked with 1% casein (ThermoFisher) or 0.5% polyvinylpyrrolidone (Sigma Aldrich) in PBS for 2 hours at room temperature. Neutrophils were loaded into the 96-well plate at 0.5 × 10^6^ neutrophils per well in Hank’s balanced salt solution containing Ca^2+^ and Mg^2+^ (HBSS^++^), and then incubated at 37 °C for 35 or 65 minutes. Following incubation, neutrophils were quantified by a plate reader for fluorescence intensity (CFSE), before and after sequential gentle washes with HBSS^++^. The number of adherent neutrophils was inspected visually by light microscopy to corroborate with plate reader signal intensity. Each group was replicated in three to six wells per independent experiment.

### Neutrophil motility and spreading on glass

Neutrophils obtained after 4-day differentiation were washed and labeled using CFSE (BioLegend). Delta T dishes (Bioptech) were coated with 2.5 µg/mL ICAM-1 and 2.5 µg/mL CXCL1 in PBS overnight at 4 °C and blocked for 1 hour with 0.5% PVP in PBS at room temperature. Approximately 75,000 neutrophils were added to warm HBSS^++^ and migration was followed using time-lapse microscopy for 30 minutes at 37 °C with images captured every 20 seconds. Motility was tracked using ImageJ Manual Tracking and analyzed using Ibidi Chemotaxis to obtain measures of migration such as accumulated distance, Euclidean distance, and velocity. Cell area was measured using the final image acquired at 30 minutes using ImageJ.

### Neutrophil spreading on polyacrylamide gels

Polyacrylamide gel substrates were prepared as originally described^50^. *See Supplementary Material*.

### Flow chamber assay

To prepare flow chambers, Ibidi µ-Slide VI^0.1^ were coated with 0.5 μg/mL E-selectin, 7 μg/mL ICAM-1, and 8 μg/mL CXCL1 for 2 hours in PBS and then blocked with an excess of casein for 2 hours, both at room temperature. Flow chambers were perfused at 12.98 μL/min, which is calculated to produce a shear stress of 1 dyne/cm^2^. CFSE-labeled wild-type and vehicle control-treated vinculin knockout neutrophils were evaluated within the same flow chamber. Time-lapse images were captured every 10 seconds for 1 hour, starting immediately after starting flow chamber perfusion, using transmitted light through a 20X objective. Motility was tracked using ImageJ Manual Tracking and analyzed using Ibidi Chemotaxis to obtain measures of migration: accumulated distance, Euclidean distance, and velocity.

### Western blot

*See Supplementary Material*.

### Immunocytochemistry and TIRF microscopy

Glass coverslips (0.17 mm) were coated with 10 μg/mL ICAM-1 and 2.5 µg/mL CXCL1 for 2 hours and then blocked with an excess of casein for 2 hours. Neutrophils obtained after 4-day differentiation were washed and resuspended in HBSS^++^ prior to use. Neutrophils were incubated on coverslips for 35 minutes at 37 °C, then fixed in 10% neutral buffered formalin for 30 minutes, and permeabilized in 0.1% Triton X-100 for 10 minutes. Coverslips were incubated with primary antibodies in 1% BSA overnight at 4 °C, transferred to secondary antibodies for 1 hour at room temperature. Neutrophils were stained with NucBlue and ActinGreen 488 (ThermoFisher Scientific) according to manufacturer’s recommendation prior to mounting coverslips onto slides. For live cell imaging, progenitors with transduced to express Clover-vinculin and Lifeact-mRuby2 in differentiated neutrophils. Samples were imaged with a TILL Photonics iMIC TIRF microscope (FEI Company) with a 60x objective with 1.49 numerical aperture (Olympus) and Andor iXon3 EMCCD camera. Image analyses were performed using ImageJ software.

### Animals

All animal studies were approved by the Lifespan Animal Welfare Committee (Approval #0089-16, Office of Laboratory Animal Welfare Assurance #A3922-01) and were performed in accordance with relevant guidelines and regulations. Mice were housed in a specific pathogen-free facility at Rhode Island Hospital. Mice harboring floxed *Vcl* alleles (Vcl^f/f^) were kindly provided by Dr. Robert Ross (UC-San Diego) and have been previously described^51^. Vcl^f/f^ mice were crossed with MX1-Cre (MX1^cre^) mice (The Jackson Laboratory) in which Cre recombinase expression is controlled by the MX1 promoter and can be induced by interferon production after administration of synthetic double-stranded RNA^52^. To generate mixed chimeras, 8- to 12-week-old C57BL/6 mice (The Jackson Laboratory) were lethally irradiated (10 Gy, single dose) and then reconstituted by intravenous injection of bone marrow cells from a Vcl^f/f^MX1^cre^GFP^+^ mouse expressing transgenic enhanced green fluorescent protein (EGFP) under the ubiquitin C promoter (The Jackson Laboratory) and a Vcl^f/f^ (GFP^-^) control mouse at 1:1 ratio. Deletion of the gene encoding vinculin was induced by intraperitoneal injection of 250 μg of polyinosinic–polycytidylic acid (Poly I:C; InvivoGen), three doses, each 2 days apart, starting 4 weeks after irradiation, inducing near complete loss of the respective protein in neutrophils (Supplementary Fig. S4). Mice were used for experiments 4–8 weeks after Poly I:C administration.

### Peritonitis model

For each mixed chimeric mouse, a blood sample was collected by saphenous vein puncture prior to intraperitoneal injection of 1 mL 4% thioglycollate broth to induce peritonitis. For adoptive transfer studies, mice were first challenged with an intraperitoneal dose of 1 mL 4% thioglycollate broth. At 2 hours post-challenge, mice were intravenously injected with a mixed 1:1 population of 6 × 10^6^ membrane dye-labeled *in vitro*-derived neutrophils. At the indicated time point, 5 mL of ice-cold PBS with 2 mM EDTA was used to lavage the peritoneum. Blood and lavage were analyzed by flow cytometry using fluorescently labeled anti-Ly6G antibody to distinguish neutrophils with a MACSQuant Analyzer 10 (Miltenyi).

### Cremaster muscle intravital imaging

Mice were anesthetized using a cocktail of ketamine (125 mg/kg) and xylazine (12.5 mg/kg), the carotid artery was cannulated, and the cremaster muscle was exteriorized, cut longitudinally, and spread onto a stage as has been previously described^6^. The cremaster muscle was perfused throughout the experiment with 37 °C bicarbonate buffered saline equilibrated with 5% CO_2_ in N_2_. A blood sample was collected prior to imaging using a catheter placed in the carotid artery. For arrest assays, 600 ng murine CXCL1 was intravenously administered through the catheter. Cremaster muscle post-capillary venules were imaged for 14 minutes following CXCL1 injection using an upright Olympus BX60 microscope with a 40X water-immersion objective with EGFP fluorescence captured by a Chameleon 3 color camera (FLIR Systems) and Rapp SP20-X3 xenon flashlamp.

### Traction force microscopy

Traction force microscopy was performed as previously described, with some modifications^53^. *See Supplementary Material*.

## Data analysis

All analyses were performed using GraphPad Prism 8, with tests for normality of distribution and for equal variance performed using Sigmaplot. As indicated, one-way or two-way analysis of variance (ANOVA) was used to compare the differences between samples and post-hoc analysis was performed using Tukey pairwise multiple comparison test. For samples that did not have a normal distribution, Mann-Whitney (two groups) or Kruskal-Wallis one-way ANOVA was used to compare difference between samples and post-hoc analysis was performed using Tukey pairwise multiple comparison test. For comparison of two groups with normally distributed data, an unpaired, two-tailed Student’s t-test was used. Experimental data are presented with mean and standard deviation. For traction force microscopy, µ is log transformed as this better describes the distribution of values.

## Supplementary information

Supplementary information.

Supplementary information 2.

## References

[1] Walzog B, Scharffetter-Kochanek K, Gaehtgens P (1999). Impairment of neutrophil emigration in CD18-null mice. Am. J. Physiol..

[2] Ley K, Laudanna C, Cybulsky MI, Nourshargh S (2007). Getting to the site of inflammation: the leukocyte adhesion cascade updated. Nat. Rev. Immunol..

[3] Anderson DC (1985). The Severe and Moderate Phenotypes of Heritable Mac-1, Lfa-1 Deficiency - Their Quantitative Definition and Relation to Leukocyte Dysfunction and Clinical-Features. J. Infect. Dis..

[4] Grommes J, Soehnlein O (2011). Contribution of neutrophils to acute lung injury. Mol. Med..

[5] Lu C (2001). An isolated, surface-expressed I domain of the integrin alphaLbeta2 is sufficient for strong adhesive function when locked in the open conformation with a disulfide bond. Proc. Natl Acad. Sci. USA.

[6] Lefort CT (2012). Distinct roles for talin-1 and kindlin-3 in LFA-1 extension and affinity regulation. Blood.

[7] Lefort CT, Ley K (2012). Neutrophil arrest by LFA-1 activation. Front. Immunol..

[8] Phillipson M (2006). Intraluminal crawling of neutrophils to emigration sites: a molecularly distinct process from adhesion in the recruitment cascade. J. Exp. Med..

[9] Pick R, Brechtefeld D, Walzog B (2013). Intraluminal crawling versus interstitial neutrophil migration during inflammation. Mol. Immunol..

[10] Peng X, Nelson ES, Maiers JL, DeMali KA (2011). New insights into vinculin function and regulation. Int. Rev. Cell Mol. Biol..

[11] Humphries JD (2007). Vinculin controls focal adhesion formation by direct interactions with talin and actin. J. Cell Biol..

[12] Atherton P (2015). Vinculin controls talin engagement with the actomyosin machinery. Nat. Commun..

[13] Propato A (2001). Apoptotic cells overexpress vinculin and induce vinculin-specific cytotoxic T-cell cross-priming. Nat. Med..

[14] Saez de Guinoa J, Barrio L, Carrasco YR (2013). Vinculin arrests motile B cells by stabilizing integrin clustering at the immune synapse. J. Immunol..

[15] Walde M, Monypenny J, Heintzmann R, Jones GE, Cox S (2014). Vinculin binding angle in podosomes revealed by high resolution microscopy. PLoS One.

[16] Fukunaga T, Zou W, Warren JT, Teitelbaum SL (2014). Vinculin regulates osteoclast function. J. Biol. Chem..

[17] Lammermann T, Sixt M (2009). Mechanical modes of ‘amoeboid’ cell migration. Curr. Opin. Cell Biol..

[18] Yuruker B, Niggli V (1992). Alpha-actinin and vinculin in human neutrophils: reorganization during adhesion and relation to the actin network. J. Cell Sci..

[19] Zhang H (2006). Impaired integrin-dependent function in Wiskott-Aldrich syndrome protein-deficient murine and human neutrophils. Immun..

[20] Wilson ZS (2017). Activated beta2 Integrins Restrict Neutrophil Recruitment during Murine Acute Pseudomonal Pneumonia. Am. J. Respir. Cell Mol. Biol..

[21] Smith LA, Aranda-Espinoza H, Haun JB, Dembo M, Hammer DA (2007). Neutrophil traction stresses are concentrated in the uropod during migration. Biophys. J..

[22] Weiner OD (1999). Spatial control of actin polymerization during neutrophil chemotaxis. Nat. Cell Biol..

[23] Cassimeris L (1990). Chemoattractant-stimulated polymorphonuclear leukocytes contain two populations of actin filaments that differ in their spatial distributions and relative stabilities. J. Cell Biol..

[24] Toyjanova J, Flores-Cortez E, Reichner JS, Franck C (2015). Matrix confinement plays a pivotal role in regulating neutrophil-generated tractions, speed, and integrin utilization. J. Biol. Chem..

[25] Lokuta MA, Huttenlocher A (2005). TNF-alpha promotes a stop signal that inhibits neutrophil polarization and migration via a p38 MAPK pathway. J. Leukoc. Biol..

[26] Bertram A (2012). Protein kinase C-theta is required for murine neutrophil recruitment and adhesion strengthening under flow. J. Immunol..

[27] Nagasato AI, Yamashita H, Matsuo M, Ueda K, Kioka N (2017). The distribution of vinculin to lipid rafts plays an important role in sensing stiffness of extracellular matrix. Biosci. Biotechnol. Biochem..

[28] del Rio A (2009). Stretching single talin rod molecules activates vinculin binding. Sci..

[29] Oakes PW (2009). Neutrophil morphology and migration are affected by substrate elasticity. Blood.

[30] Janostiak R (2014). CAS directly interacts with vinculin to control mechanosensing and focal adhesion dynamics. Cell Mol. Life Sci..

[31] Gautrot JE (2014). The nanoscale geometrical maturation of focal adhesions controls stem cell differentiation and mechanotransduction. Nano Lett..

[32] Nolz JC (2007). WAVE2 regulates high-affinity integrin binding by recruiting vinculin and talin to the immunological synapse. Mol. Cell Biol..

[33] Mitsios JV (2010). What is vinculin needed for in platelets?. J. Thromb. Haemost..

[34] Takubo T, Tatsumi N (1999). Microscopic co-distributions of myosin, actin, alpha-actinin and vinculin in human neutrophils during movement. Haematol..

[35] Massena S (2010). A chemotactic gradient sequestered on endothelial heparan sulfate induces directional intraluminal crawling of neutrophils. Blood.

[36] Hepper I (2012). The mammalian actin-binding protein 1 is critical for spreading and intraluminal crawling of neutrophils under flow conditions. J. Immunol..

[37] Alcaide P, Auerbach S, Luscinskas FW (2009). Neutrophil recruitment under shear flow: it’s all about endothelial cell rings and gaps. Microcirculation.

[38] McDonald B (2010). Intravascular danger signals guide neutrophils to sites of sterile inflammation. Sci..

[39] Rahman Aniqua, Carey Shawn P., Kraning-Rush Casey M., Goldblatt Zachary E., Bordeleau Francois, Lampi Marsha C., Lin Deanna Y., García Andrés J., Reinhart-King Cynthia A. (2016). Vinculin regulates directionality and cell polarity in two- and three-dimensional matrix and three-dimensional microtrack migration. Molecular Biology of the Cell.

[40] Qian H, Sheetz MP, Elson EL (1991). Single particle tracking. Analysis of diffusion and flow in two-dimensional systems. Biophys. J..

[41] Mocsai A (2006). Integrin signaling in neutrophils and macrophages uses adaptors containing immunoreceptor tyrosine-based activation motifs. Nat. Immunol..

[42] Futosi K, Mocsai A (2016). Tyrosine kinase signaling pathways in neutrophils. Immunol. Rev..

[43] Volmering S, Block H, Boras M, Lowell CA, Zarbock A (2016). The Neutrophil Btk Signalosome Regulates Integrin Activation during Sterile Inflammation. Immun..

[44] Lammermann T (2013). Neutrophil swarms require LTB4 and integrins at sites of cell death *in vivo*. Nat..

[45] Huveneers S (2012). Vinculin associates with endothelial VE-cadherin junctions to control force-dependent remodeling. J. Cell Biol..

[46] Trichet L (2012). Evidence of a large-scale mechanosensing mechanism for cellular adaptation to substrate stiffness. Proc. Natl Acad. Sci. USA.

[47] Wang GG (2006). Quantitative production of macrophages or neutrophils *ex vivo* using conditional Hoxb8. Nat. Methods.

[48] Salmanidis M (2013). Hoxb8 regulates expression of microRNAs to control cell death and differentiation. Cell Death Differ..

[49] Gurzeler U (2013). *In vitro* differentiation of near-unlimited numbers of functional mouse basophils using conditional Hoxb8. Allergy.

[50] Pelham RJ, Wang Y (1997). Cell locomotion and focal adhesions are regulated by substrate flexibility. Proc. Natl Acad. Sci. USA.

[51] Zemljic-Harpf AE (2007). Cardiac-myocyte-specific excision of the vinculin gene disrupts cellular junctions, causing sudden death or dilated cardiomyopathy. Mol. Cell Biol..

[52] Velasco-Hernandez T, Sawen P, Bryder D, Cammenga J (2016). Potential Pitfalls of the Mx1-Cre System: Implications for Experimental Modeling of Normal and Malignant Hematopoiesis. Stem Cell Rep..

[53] Toyjanova J (2014). High resolution, large deformation 3D traction force microscopy. PLoS One.

