# Supplementary material for: Context-Dependent Role of Vinculin in Neutrophil Adhesion, Motility and Trafficking
Source: Sci Rep. 2020 Feb 7;10:2142. doi: 10.1038/s41598-020-58882-y (PMC7005776; doi:10.1038/s41598-020-58882-y)
Supplement: Supplementary file 1 — Supplementary information. [file 41598_2020_58882_MOESM1_ESM.pdf]

Supplementary Materials

Context-dependent role of vinculin in neutrophil adhesion, motility and trafficking

Zachary S. Wilson[1,2], Hadley Witt[1,2], Lauren Hazlett[3], Michael Harman[2,3], Brittany M. Neumann[2], Andrew Whitman[2], Mohak Patel[3], Robert S. Ross[4], Christian Franck[5], Jonathan S. Reichner[2], Craig T. Lefort[2,*]

Supplementary Methods

Western blot

For each group, $1x10^6$ neutrophils were lysed in Tris lysis buffer (0.2M Tris, 1% Triton X-100, 1% sodium orthovandate) containing protease inhibitor cocktail (Sigma Aldrich) for 30 minutes on ice with occasional mixing. Lysed samples were centrifuged at 11,000g for 10 minutes and the supernatants were heated to 95˚C for 10 minutes in Laemmli buffer. Supernatants were loaded onto 4-20% Mini-PROTEAN TGX gels (Bio-Rad) and run at 160V. Gels were then transferred onto 0.45 µm nitrocellulose membrane at 100V for 1 hour at 4˚C. Probing of western blots was carried out according to the antibody manufacturer's instructions (Cell Signaling Technologies). Western blots were analyzed with SuperSignal West Pico PLUS Chemiluminescent Substrate (Thermo Scientific) to detect HRP-conjugated antibodies using a Bio-Rad Chemidoc XRS.

Neutrophil spreading on polyacrylamide gels

Polyacrylamide gel substrates were prepared as originally described [1]. Gels were prepared using varying concentrations of acrylamide (Bio-Rad) and *N,N*-methylene-bisacrylamide (Bio-Rad) to achieve the range of elasticity. Stiff substrates were chosen to be 12% acrylamide and 0.4% bisacrylamide, at approximately 100 kPa stiffness; intermediate stiffness substrates were chosen to be 8%/0.08%, at approximately 8.3 kPa; and soft substrates were chosen to be 3%/0.2%, at approximately 1.5 kPa. Within the smaller range of intermediate stiffness gels, acrylamide/bisacrylamide used were: 6.4%/0.23% for approximately 20 kPa stiffness, 5.2%/0.19% for approximately 10 kPa, and 4.4%/0.16% for approximately 5 kPa. Polyacrylamide solutions were vortexed and then polymerized through the addition of excess tetramethylethylenediamine and ammonium persulfate. Gels were polymerized at room temperature on hydrophilic-treated, glass-bottom deltaT dishes (Bioptech) and shaped using AbGene frames to a final size of approximately 1 cm x 1 cm x 300 µm. Gels were then soaked for at least one hour in water to remove any unpolymerized acrylamide. Final elasticity was measured by atomic force

microscopy. Sulfo-SANPAH was allowed to covalently bond to the gel for 1 hour in the dark. ICAM-1 and/or CXCL1 were then UV cross-linked to the sulfo-SANPAH at 2.5 μg/mL each. Previous work shows that final elasticity is not affected by surface protein crosslinking, and that final protein density is not affected by elasticity [2]. After washing with buffered saline, *in vitro*-derived neutrophils were added in warmed $HBSS^{++}$ and incubated at 37°C. Imaging was performed 30 minutes after incubation. For motility assays, imaging was performed within a fully enclosed microscope at 37°C for 30 minutes in L-15 media supplemented with 2 mg/ml glucose.

Traction force microscopy

Traction force microscopy was performed as previously described, with some modifications [3]. Briefly, neutrophils obtained after 4-day differentiation were washed, labeled using CFSE (ThermoFisher Scientific), and resuspended in $HBSS^{++}$. Polyacrylamide gels were prepared using varying concentrations of acrylamide (Bio-Rad) and *N,N*-methylene-bisacrylamide (Bio-Rad) to achieve the range of desired elasticities with a Poisson's ratio of 0.5. Soft gel substrates were composed of 3% acrylamide and 0.2% bisacrylamide at approximately 1.5kPa, very soft substrates were composed of 3% acrylamide and 0.06% bisacrylamide at approximately 0.5 kPa. Gel substrates were embedded with 0.2% final w/v 0.5 μm fluorescent microspheres (Invitrogen). Polyacrylamide gels were crosslinked using 1.25% APS and 0.5% TEMED yielding a final gel thickness of approximately 40 μm. 100 μg/mL ICAM-1 and 5 μg/mL CXCL1 were UV cross-linked to the sulfo-SANPAH, as described above. Three-dimensional time-lapse volumetric images of fluorescent beads in polyacrylamide substrates and labeled cell membranes were recorded using laser scanning confocal microscopy (LSCM). A fast iterative digital volume correlation (FIDVC) algorithm was used to track the motion of beads and calculate the 3D displacement vector during cell migration compared to a reference position obtained by detaching the cells using 2% sodium dodecyl sulfate (Sigma Aldrich) [3].

To calculate cell tractions, $\mathbf{T}$, and an overall measure of cellular contractility, μ, the Cauchy stress, $\boldsymbol{\sigma}$, was determined using the following equation for a Neo-Hookean elastic solid,

$$\boldsymbol{\sigma} = \frac{G}{J^{5/3}}\left(\mathbf{B} - \frac{1}{3}\mathrm{tr}(\mathbf{B})\,\mathbf{I}\right) + K\,(J-1)\mathbf{I}.$$

Here, J is the Jacobian of the deformation gradient tensor, $\mathbf{B}$ is the left Cauchy-Green deformation tensor, and G and K are the shear and bulk modulus of the polyacrylamide gel, respectively. To compute the surface normal, $\mathbf{n}$, we applied the Delaunay triangulation on the deformed surface grid points. The reference gel surface is assumed to be initially flat in the stress-free-state, and we compute the deformed surface grid points by adding the cumulative surface displacement to the reference gel surface.

Using the surface normal, $\mathbf{n}$, and the Cauchy stress, $\boldsymbol{\sigma}$, we compute the cell tractions, $\mathbf{T}$, and root-mean squared traction, T_{rms}, as follows,

$$\mathbf{T} = \mathbf{n}\cdot\boldsymbol{\sigma} \qquad T_{rms} = \sqrt{\frac{1}{N}\sum_{i=1}^{N}\mathbf{T}_i^2}.$$

To establish a simple, scalar-based metric of the overall contractility of the cell, we extended the general procedure by Butler et al. to calculate the three-dimensional dipole moment tensor as,

$$\boldsymbol{M} = \int_S \boldsymbol{x}\boldsymbol{T}\,dS\,.$$

The cellular contractility, μ, is then defined simply as the trace of the dipole moment tensor $\mathbf{M}$, i.e., $\mu = \mathrm{tr}(\mathbf{M})$ [4].

Supplementary Figure Legends

Supplementary Video 1. Neutrophil migration in a flow chamber. Flow chambers coated with E-selectin, ICAM-1, and CXCL1 were perfused for 30 minutes with a 1:1 mix of WT (CFSE labeled) and VclKO neutrophils at a wall shear stress of 1 dyne/cm^2. Scale bar = 10 μm.

Figure S1. **Differentiated HoxB8-conditional progenitors display a neutrophil phenotype.** (A) Schematic of the inducible HoxB8 progenitor system and G-CSF-induced differentiation into neutrophils. (B) Wright-Giemsa stain of *in vitro* progenitor-derived neutrophils. (C) Flow cytometry of day 4 differentiated progenitors, analyzing neutrophil marker Ly6G and progenitor marker CD117.

Figure S2. Knockout of vinculin in HoxB8-conditional progenitors and neutrophils. (A) Schematic of CRISPR/Cas9 targeting of the murine *Vcl* locus with independent sgRNAs. (B) Representative western blot of *in vitro*-derived neutrophils, derived from progenitor lines expressing Cas9 and targeting or non-targeting unrelated (UR) sgRNA. Blots were probed with antibodies to measure protein expression of vinculin and loading control α-actinin. (C) Representative flow cytometry histograms of wild-type (WT) and vinculin knockout (VclKO) progenitors and neutrophils, analyzing HoxB8-GFP expression (representative of 3 independent experiments).

Figure S3. Exogenous re-expression of Clover-vinculin. Representative western blot of *in vitro*-derived neutrophil protein expression of vinculin (endogenous, recognized by anti-vinculin), Clover-vinculin (exogenous, recognized by both anti-vinculin and anti-GFP), and loading control α-actinin. Clover-vinculin is abbreviated CV.

Figure S4. Tissue-specific knockout of vinculin in mice. Representative western blot of purified bone marrow neutrophils from control, $Vcl^{f/f}$, and $Vcl^{f/f}MX1^{cre}$ mice, with and without Poly I:C activation of MX1-Cre recombinase-induced disruption of *Vcl*. Western blots were probed for protein expression of vinculin and loading control α-actinin.

Figure S5. Expression of adhesion and signaling receptors in progenitor-derived neutrophils. Flow cytometry quantification of cell surface expression of (A) CD11a, (B) CD11b, and (C) CXCR2 by mean fluorescent intensity (MFI). Groups of *in vitro*-derived neutrophils are indicated and are shown with analyses of bone marrow neutrophils (n=4 independent experiments, except for BM where n=2). (D) Flow cytometry analysis of CD11b upregulation after fMLP stimulation for WT and VclKO neutrophils (representative of n=2 experiments).

Figure S6. Additional analyses of neutrophil static adhesion assays. (A-C) Normalized adhesion of CFSE-labeled neutrophils under conditions as indicated: (A) Evaluation of CXCL1-induced neutrophil adhesion to ICAM-1, (B) Analysis of sequential washes to remove non-adherent neutrophils, (C) Analysis of coating concentration dependence of neutrophil adhesion. All experiments were analyzed using one-way or two-way ANOVA with Tukey multiple comparison test (3 independent experiments). * $p<0.05$; ** $p<0.01$; *** $p<0.001$; **** $p<0.0001$.

Figure S7. Vinculin-deficient neutrophils display a less polarized phenotype. (A) Representative image of F-actin distribution (by TIRFM) of WT and VclKO neutrophils, displayed with a cross-sectional histogram of fluorescence intensity at the plane indicated. (B) Analyses of the fluorescent distribution of

actin and shape of polarized neutrophils based on F-actin staining of neutrophil after 30 minute adhesion and spreading on ICAM-1 and CXCL1. Data were analyzed using Kruskal-Wallis one-way ANOVA on ranks with Dunn's multiple comparison test (n>30 cells per group, 3 independent experiments). * $p<0.05$. ** $p<0.01$. *** $p<0.001$, **** $p<0.0001$.

Figure S8. Vinculin localizes to contracting pseudopods. (A) Migration of neutrophils expressing Clover-vinculin and Lifeact-mRuby2 on a substrate of ICAM-1 and CXCL1 over the course of 10 minutes (representative of 3 independent experiments). Arrows point to punctate vinculin-containing structures. Scale bar = 10 μm. (B) Immunocytochemistry of neutrophils fixed after 30 minutes of migration on ICAM-1 and CXCL1. Samples were stained with antibodies against vinculin and CD11a; phalloidin and Hoescht used for F-actin and nuclear staining. Scale bar = 10 μm.

Figure S9. Analyses of neutrophil trafficking during sterile peritonitis. (A) Schematic that depicts the experimental design for analyzing neutrophil trafficking in murine mixed chimeras reconstituted with both control ($Vcl^{f/f}$) and vinculin knockout ($Vcl^{f/f}MX1^{cre}GFP^{+}$) bone marrow. Induction of vinculin knockout was achieved with Poly I:C treatment. (B) Representative flow cytometry dot plots of peripheral blood and peritoneal lavage of mixed chimeric mice, depicting the method to determine the fraction of wild-type and vinculin knockout neutrophils within those compartments. (C-D) Experiments to determine the trafficking of progenitor-derived neutrophils that were labeled with distinct fluorescent dyes, mixed in 1:1 ratios, and intravenously injected into mice during the course of thioglycollate-induced peritonitis. In (C), representative (top) and composite (bottom) analysis of the frequency of adoptively transplanted TagIt-Violet labeled WT neutrophils (*in vitro*-derived) and CFSE labeled VclKO neutrophils (*in vitro*-derived) in the blood and peritoneal lavage, 4 hours after induction of peritonitis. In (D), representative analysis of the frequency of adoptively transplanted PKH67 labeled WT neutrophils (*in vitro*-derived) and

PKH26 labeled Itgb2KO neutrophils (*in vitro*-derived) in the blood and peritoneal lavage, 4 hours after induction of peritonitis. As a control, these same analyses are shown for mice receiving intraperitoneal saline rather than 4% thioglycollate.

Figure S10. Additional analyses of neutrophil mechanosensing. (A) Representative images of WT, VclKO, and Itgb2KO neutrophil spreading on ICAM-1/CXCL1-conjugated polyacrylamide gels across a range of stiffness, as imaged using a 40X objective, DIC light microscope. Scale bar = 10 μm. (B, C) Further analyses of WT and VclKO neutrophil spread area and motility parameters (accumulated distance, Euclidean distance, velocity) on ICAM-1/CXCL1-conjugated polyacrylamide gels of intermediate stiffnesses: 5 kPa, 10 kPa, and 20 kPa.

References

1 Pelham, R. J., Jr. & Wang, Y. Cell locomotion and focal adhesions are regulated by substrate flexibility. *Proc Natl Acad Sci U S A* **94**, 13661-13665 (1997).

2 Oakes, P. W. *et al.* Neutrophil morphology and migration are affected by substrate elasticity. *Blood* **114**, 1387-1395, doi:10.1182/blood-2008-11-191445 (2009).

3 Toyjanova, J. *et al.* High resolution, large deformation 3D traction force microscopy. *PLoS One* **9**, e90976, doi:10.1371/journal.pone.0090976 (2014).

4 Butler, J. P., Tolic-Norrelykke, I. M., Fabry, B. & Fredberg, J. J. Traction fields, moments, and strain energy that cells exert on their surroundings. *Am J Physiol Cell Physiol* **282**, C595-605, doi:10.1152/ajpcell.00270.2001 (2002).

Figure S1

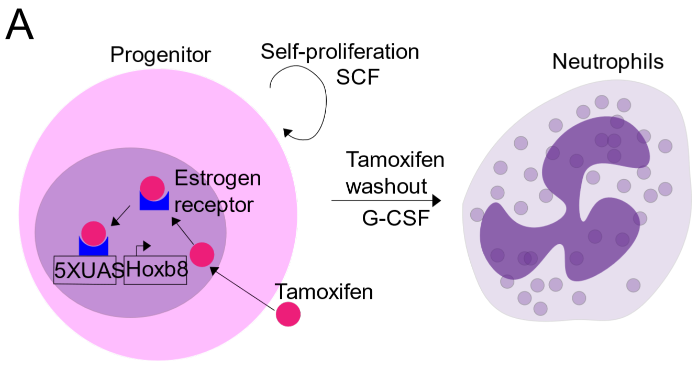
A
Progenitor
Self-proliferation
SCF
Estrogen
receptor
5XUAS
Hoxb8
Tamoxifen
Tamoxifen
washout
G-CSF
Neutrophils

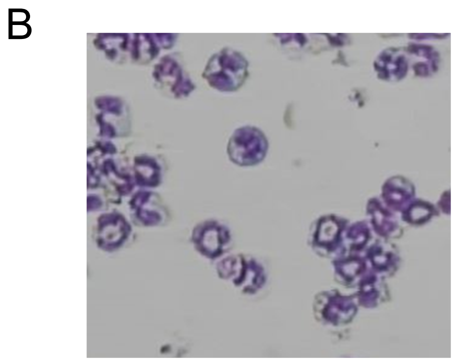
B

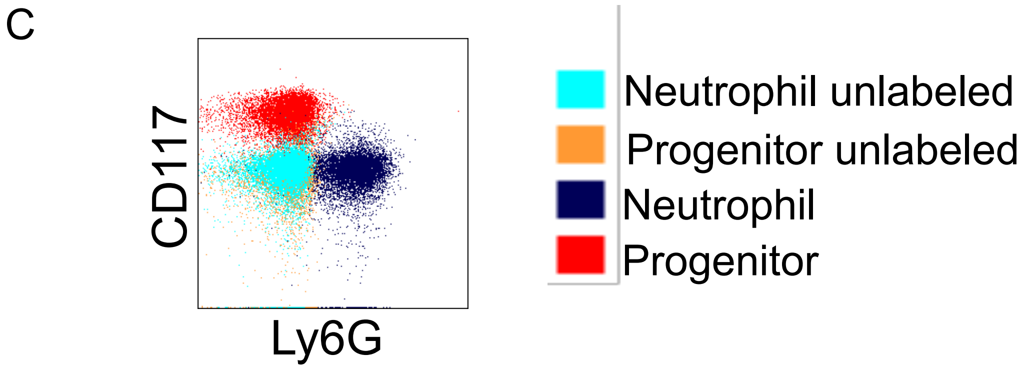
C
CD117
Ly6G
Neutrophil unlabeled
Progenitor unlabeled
Neutrophil
Progenitor

Figure S2

A

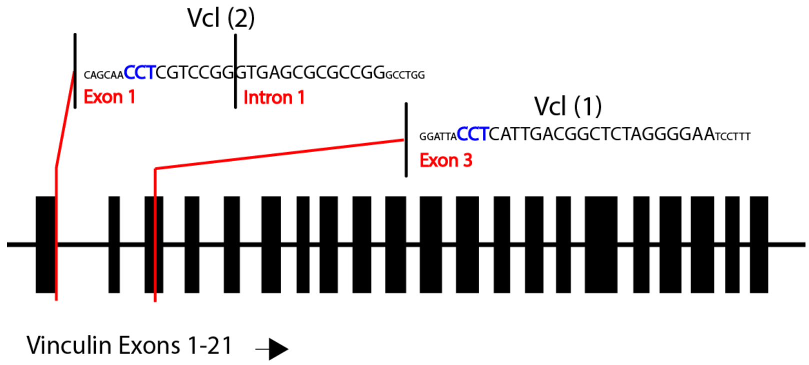

B

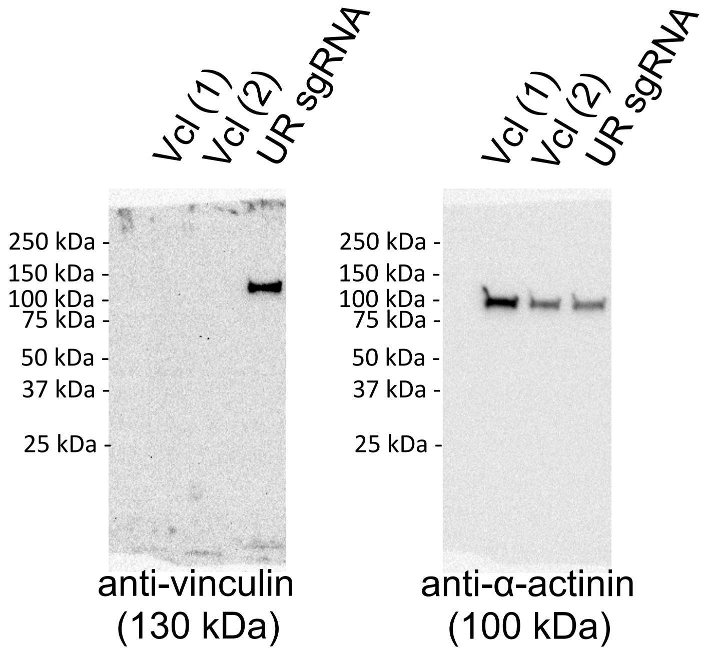

C

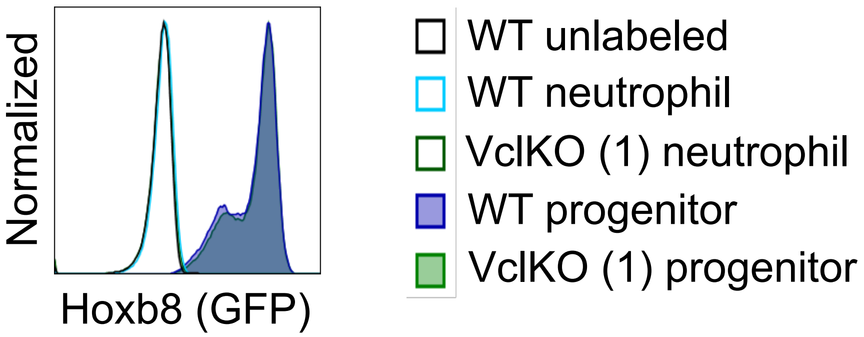

Figure S3

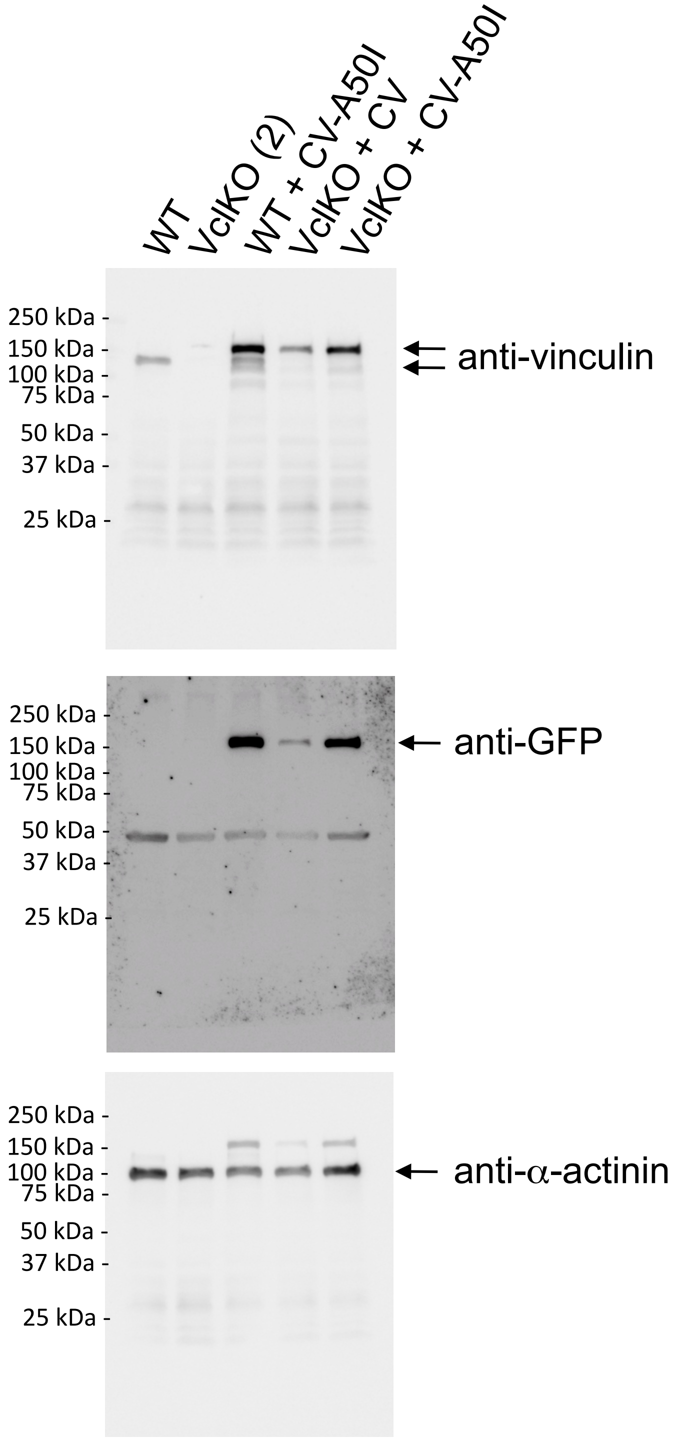
WT
VclKO (2)
WT + CV-A50I
VclKO + CV
VclKO + CV-A50I
250 kDa
150 kDa
100 kDa
75 kDa
50 kDa
37 kDa
25 kDa
anti-vinculin
anti-GFP
anti-α-actinin

Figure S4

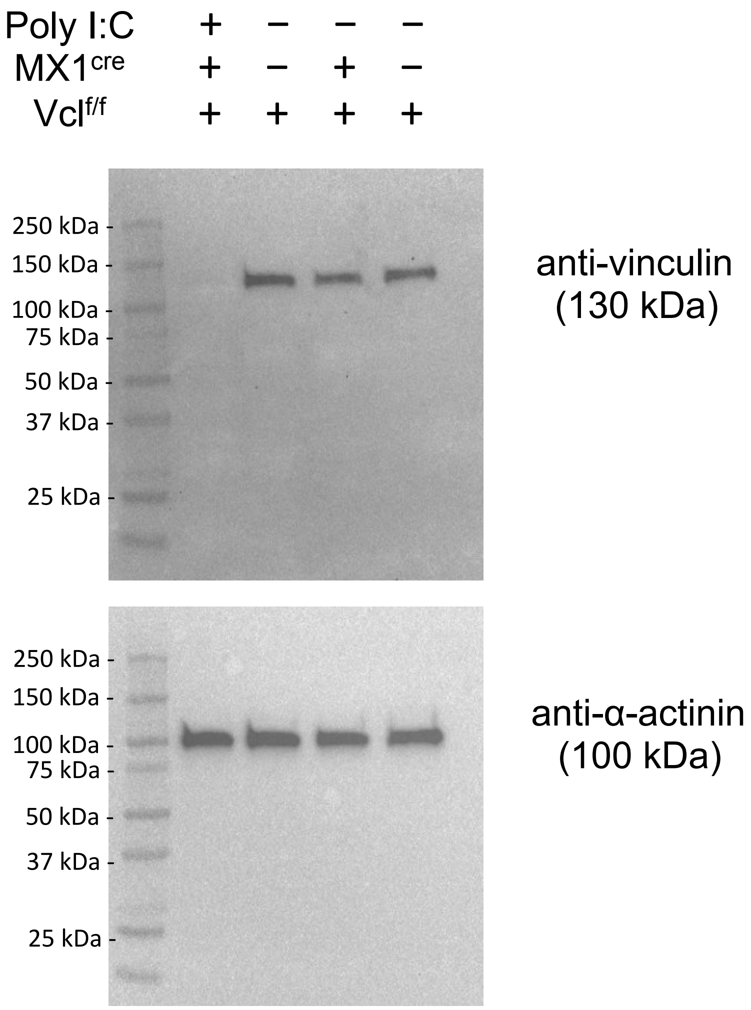
Poly I:C + − − −
MX1cre + − + −
Vclf/f + + + +
250 kDa
150 kDa
100 kDa
75 kDa
50 kDa
37 kDa
25 kDa
anti-vinculin
(130 kDa)
250 kDa
150 kDa
100 kDa
75 kDa
50 kDa
37 kDa
25 kDa
anti-α-actinin
(100 kDa)

Figure S5

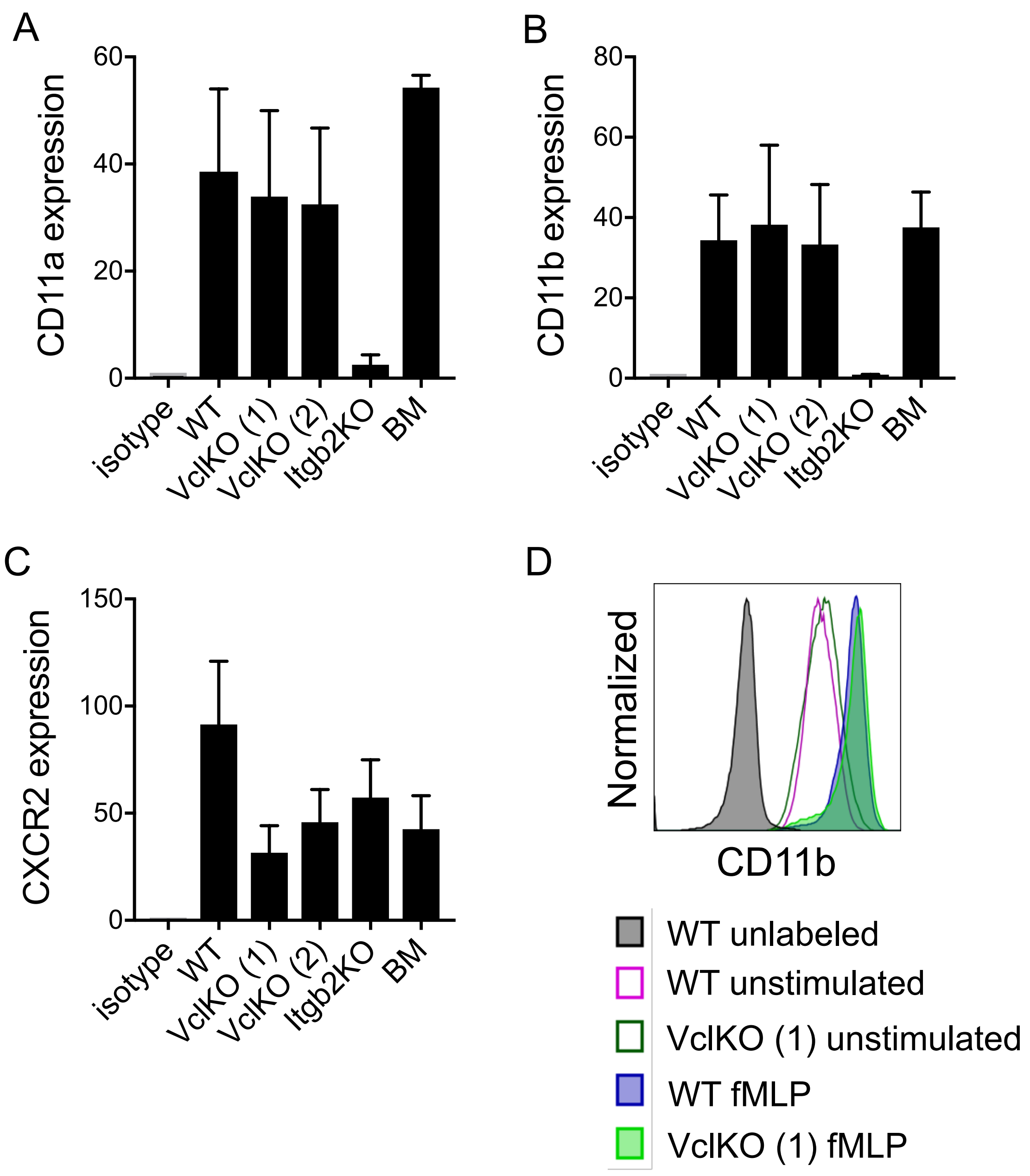
A
CD11a expression
60
40
20
0
isotype
WT
VclKO (1)
VclKO (2)
Itgb2KO
BM
B
CD11b expression
80
60
40
20
0
isotype
WT
VclKO (1)
VclKO (2)
Itgb2KO
BM
C
CXCR2 expression
150
100
50
0
isotype
WT
VclKO (1)
VclKO (2)
Itgb2KO
BM
D
Normalized
CD11b
WT unlabeled
WT unstimulated
VclKO (1) unstimulated
WT fMLP
VclKO (1) fMLP

Figure S6

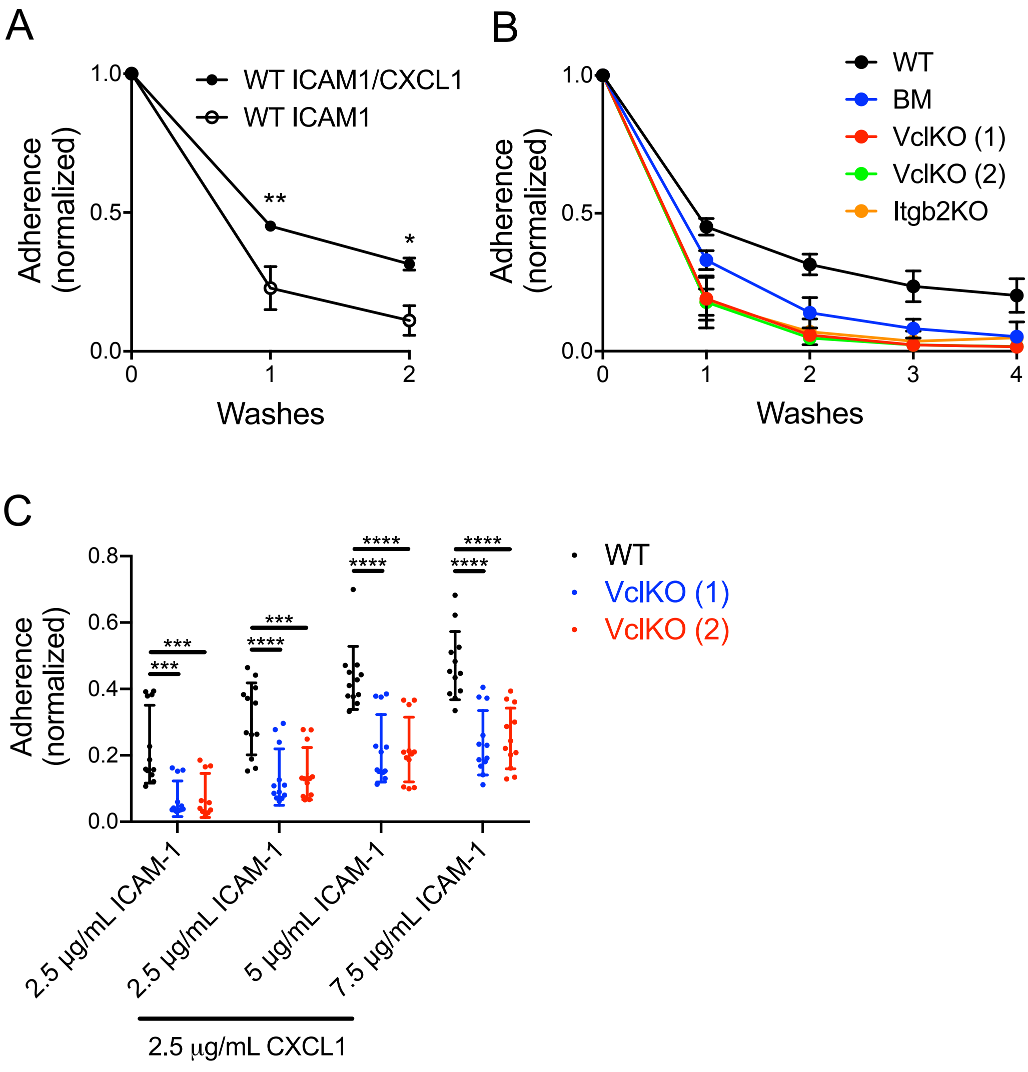

A
1.0
0.5
0.0
Adherence (normalized)
0
1
2
Washes
WT ICAM1/CXCL1
WT ICAM1
**
*
B
1.0
0.5
0.0
Adherence (normalized)
0
1
2
3
4
Washes
WT
BM
VclKO (1)
VclKO (2)
Itgb2KO
C
0.8
0.6
0.4
0.2
0.0
Adherence (normalized)
***
***
****
***
****
****
****
****
2.5 µg/mL ICAM-1
2.5 µg/mL ICAM-1
5 µg/mL ICAM-1
7.5 µg/mL ICAM-1
2.5 µg/mL CXCL1
WT
VclKO (1)
VclKO (2)

Figure S7

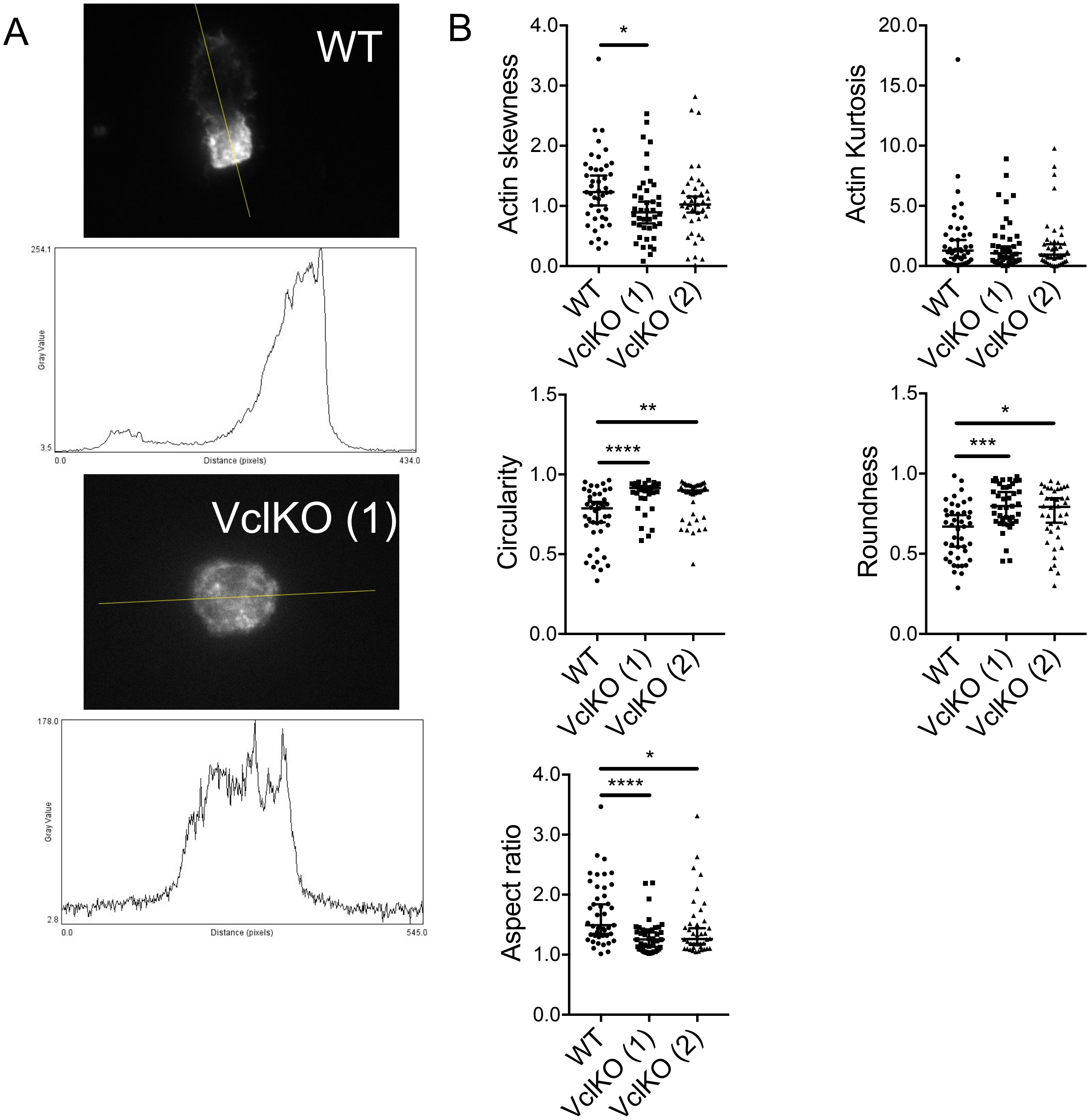
A
WT
Gray Value
Distance (pixels)
254.1
3.5
0.0
434.0
VclKO (1)
Gray Value
Distance (pixels)
178.0
2.8
0.0
545.0
B
Actin skewness
Actin Kurtosis
Circularity
Roundness
Aspect ratio
WT
VclKO (1)
VclKO (2)
*
**
***
****

Figure S8

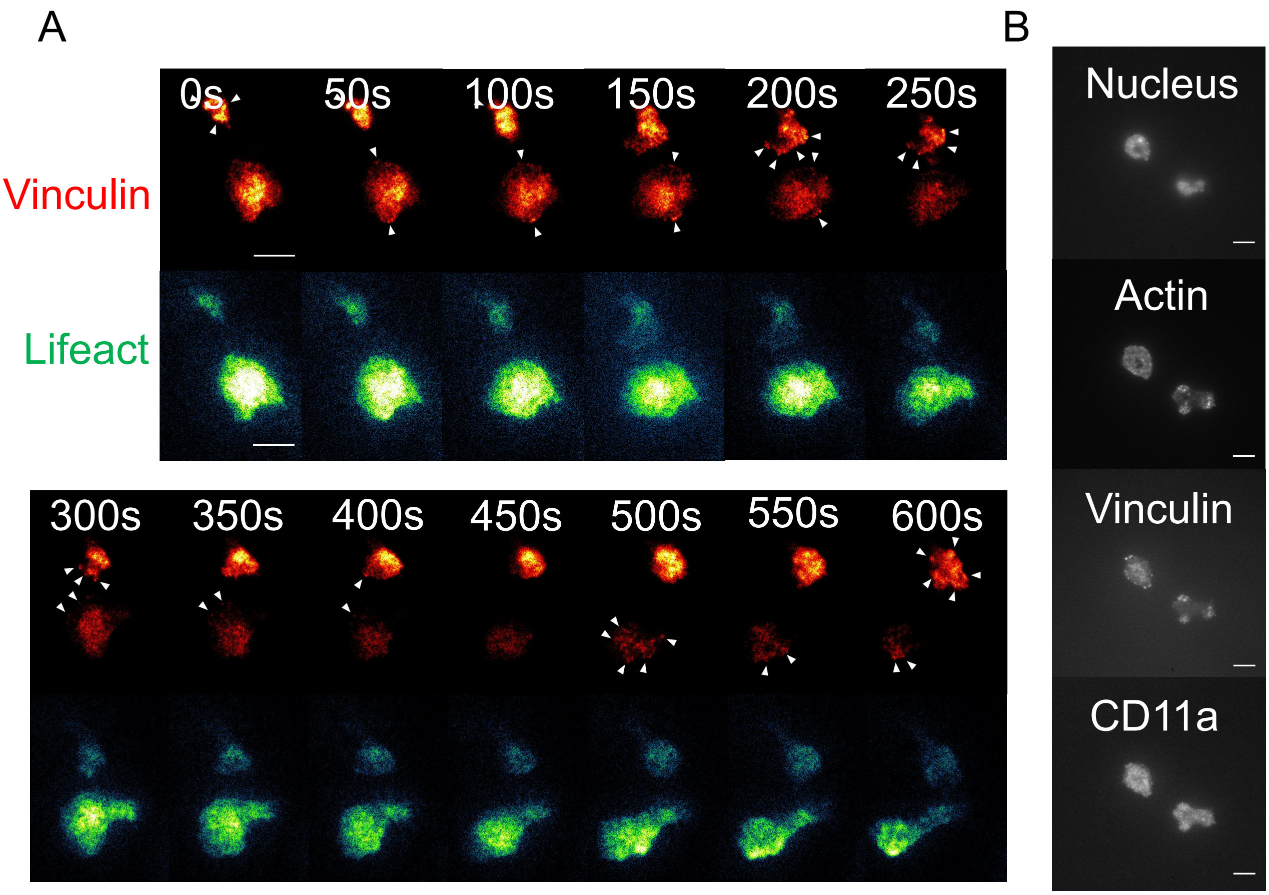
A
B
0s
50s
100s
150s
200s
250s
Vinculin
Lifeact
300s
350s
400s
450s
500s
550s
600s
Nucleus
Actin
Vinculin
CD11a

Figure S9

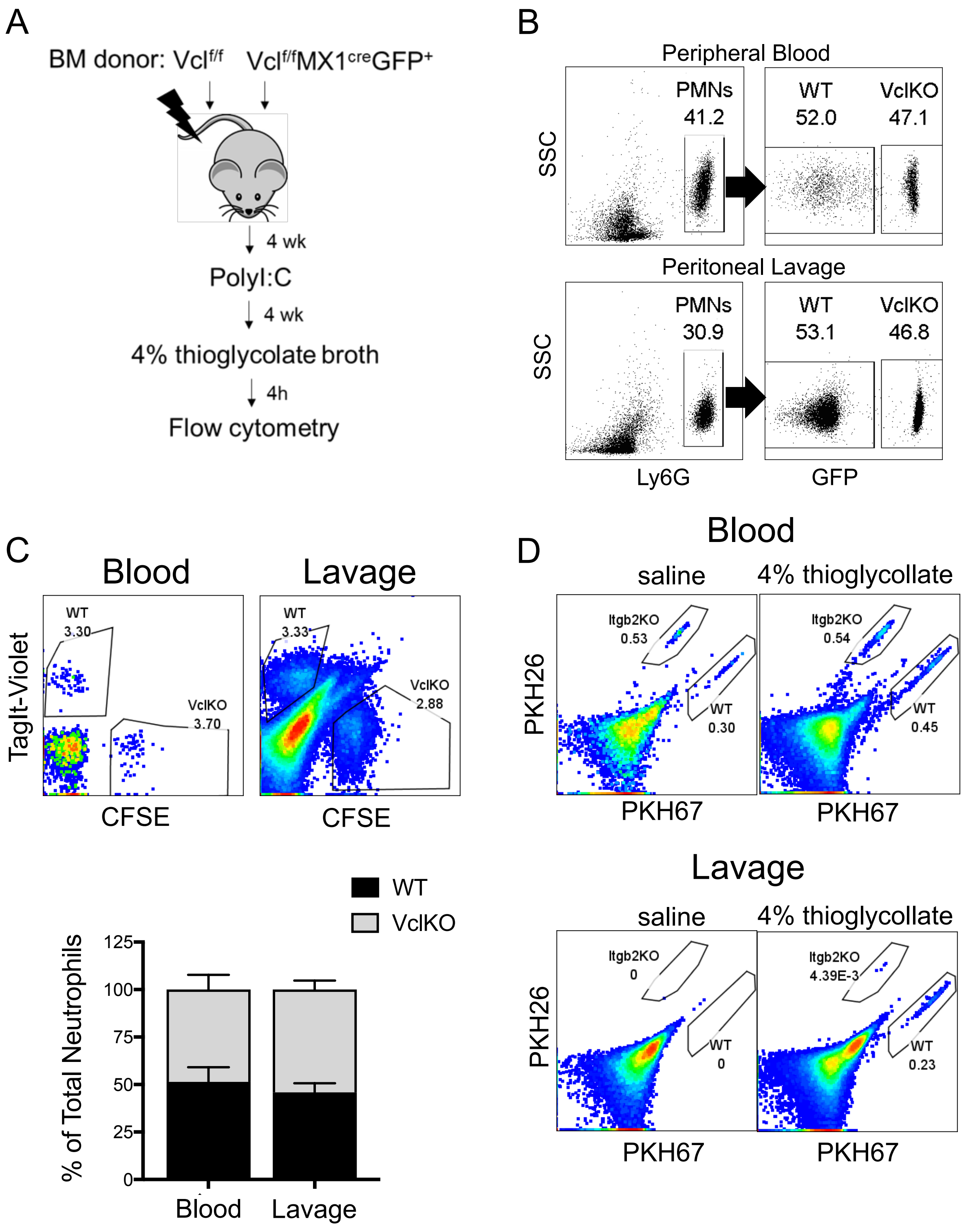
A
BM donor: Vcl$^{f/f}$ Vcl$^{f/f}$MX1^{cre}GFP^{+}
4 wk
PolyI:C
4 wk
4% thioglycolate broth
4h
Flow cytometry
B
Peripheral Blood
PMNs 41.2
WT 52.0
VclKO 47.1
Peritoneal Lavage
PMNs 30.9
WT 53.1
VclKO 46.8
SSC
Ly6G
GFP
C
Blood
Lavage
WT 3.30
VclKO 3.70
WT 3.33
VclKO 2.88
Taglt-Violet
CFSE
WT
VclKO
% of Total Neutrophils
0
25
50
75
100
125
Blood
Lavage
D
Blood
saline
4% thioglycollate
Itgb2KO 0.53
WT 0.30
Itgb2KO 0.54
WT 0.45
PKH26
PKH67
Lavage
saline
4% thioglycollate
Itgb2KO 0
WT 0
Itgb2KO 4.39E-3
WT 0.23
PKH26
PKH67

Figure S10

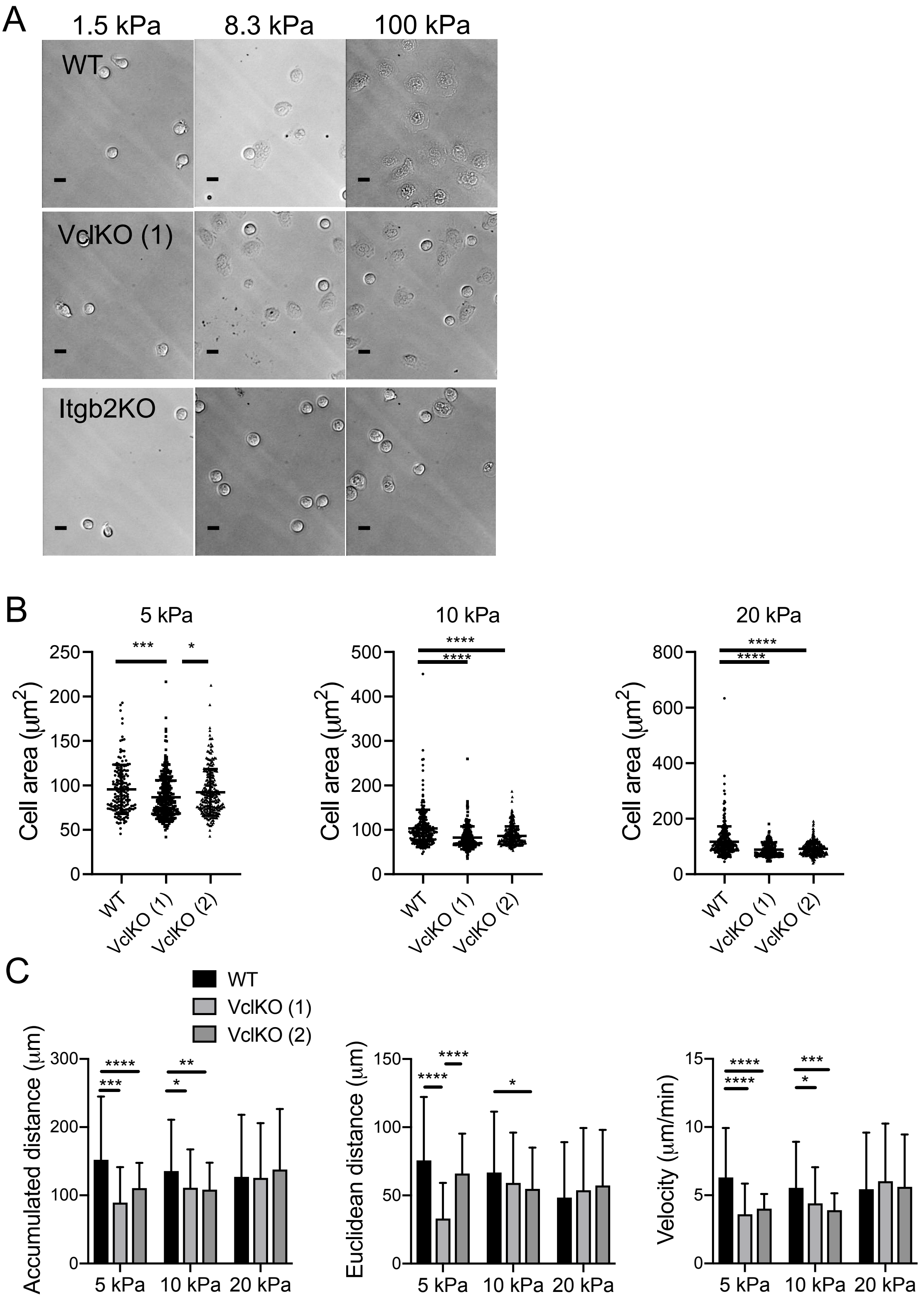

A
1.5 kPa
8.3 kPa
100 kPa
WT
VclKO (1)
Itgb2KO
B
5 kPa
10 kPa
20 kPa
Cell area (μm²)
WT
VclKO (1)
VclKO (2)
C
WT
VclKO (1)
VclKO (2)
Accumulated distance (μm)
Euclidean distance (μm)
Velocity (μm/min)
5 kPa
10 kPa
20 kPa
